# Ratiometric Chemosensors That Are Capable of Quantifying Hydrostatic Pressure Stimulus: A Case of Porphyrin Tweezers

**DOI:** 10.1021/acsphyschemau.4c00025

**Published:** 2024-07-12

**Authors:** Seiya Ono, Tomokazu Kinoshita, Hiroshi Iwasaki, Yoshitane Imai, Gaku Fukuhara

**Affiliations:** † Department of Chemistry, Tokyo Institute of Technology, 2-12-1 Ookayama, Meguro-ku, Tokyo 152-8551, Japan; ‡ Department of Applied Chemistry, Graduate School of Science and Engineering, Kindai University, 3-4-1 Kowakae, Higashi-Osaka, Osaka 577-8502, Japan

**Keywords:** hydrostatic pressure, chemosensor, porphyrin tweezer, S_2_ fluorescence, chirality, CPL

## Abstract

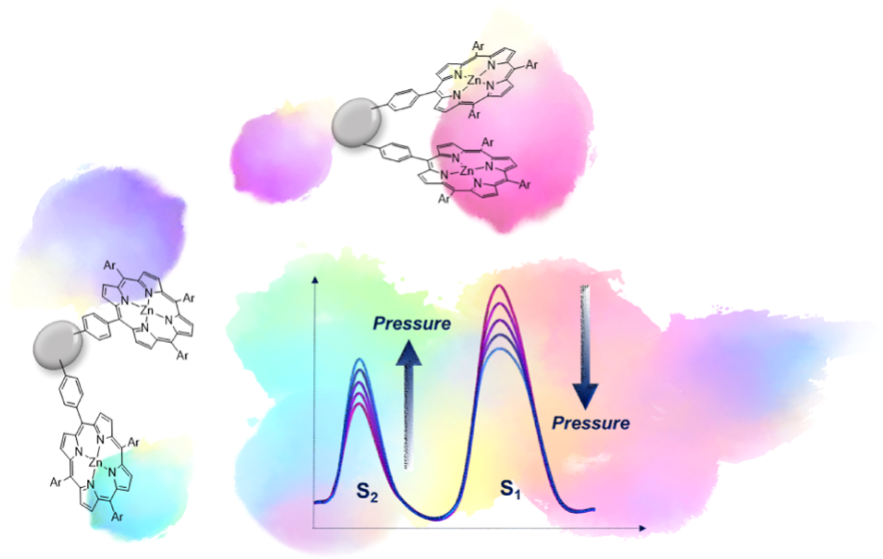

Investigating chemosensors that are capable of quantifying pressure in solution, particularly hydrostatic pressure, which is one of the mechanical forces, is an attractive challenge in chemistry from the viewpoint of “mechano”-science. Herein, we report the investigation of chiral porphyrin tweezers, **Por-Cy** and **Por-DPhEt**, comprising different flexible linkers; **Por-Cy** and **Por-DPhEt** displayed distinct ratiometric signaling by using the higher excited S_2_ state with a standard excited S_1_ level. A novel operative mechanism using the S_1_/S_2_ fluorescence ratio was revealed using hydrostatic pressure-ultraviolet/visible (UV/vis), fluorescence/excitation, circular dichroism spectroscopy, and lifetime measurements, which can be further controlled by the open-closed conformational change inherent in the tweezer skeleton. Furthermore, the fluorescent chiral tweezers exhibited a promising |*g*_lum_| of 2.9 × 10^–3^, indicating that they are potential candidates for sensory applications in chiral environments. This study provides opportunities for the development of smart pressure-responsive chemosensors.

## Introduction

The creation of chemosensors, e.g., synthetic molecules and luminescent probes, that exhibit optical and electrochemical signals in response to a wide variety of external stimuli such as pH, temperature, light excitation, solvation/viscosity, pressure, and mechano-stimuli (tension and stress), is desirable in current multidisciplinary chemistry.^1−7^ Such smart chemosensors should lead to practical applications in interfacial imaging, probing, and sensing.^8−10^ In general, using dual fluorescence chemosensors, that is, ratiometric signaling, is preferable to using sensors which have a simple on/off signaling output, when subjected to the above-mentioned stimuli.^11−18^ This preference may be attributed to the fact that the ratiometric approach is superior to the conventional approach owing to the suppression of background noises and various interferences, thereby enabling accurate imaging of living systems that contain diverse contaminants.^19−22^ In such biological systems, the fluorescence resonance energy transfer (FRET) mechanism, in which dual fluorescence in donor and acceptor chromophores mutually changes, is well applied.^9,23−25^ Hence, exploring novel dual fluorescence/ratiometric signaling chemosensors has become a focal point in research fields that necessitate new imaging/probing/sensing materials.^8−25^

Recently, hydrostatic pressure, one of the mechanical forces, has attracted considerable attention, because it is unclear how and to what extent hydrostatic pressure stimuli affect materials and biological systems in the solution state within the MPa order of pressure. These questions have been posed in the recently developed “mechano” sciences^26^ that include mechanochemistry^27−29^ and mechanobiology.^30−32^ High-pressure solid chemistry using diamond anvil cell (∼GPa) may be excluded at this point.^33,34^ Chemosensors that quantify hydrostatic pressures in a medium, that is, pressure-responsive chemosensors are required. Although the solution-state effects of hydrostatic pressure have been studied since the 1960s,^35−50^ the molecules used previously to respond to hydrostatic pressure were not suitable for quantifying the stimulus. This lack is due to the shadowgraph effect^51^ that refracts light toward a high-pressure medium. A ratiometric signaling approach that can cancel the unwanted shadowgraph effect may be a possible solution. For example, fluorophores that exhibit changes in dual fluorescence upon hydrostatic pressurization have been developed using excimer^36,39^ and twisted intramolecular charge transfer (TICT)^40,52^ mechanisms. Based on ratiometric signaling guidelines, we synthesized hydrostatic-pressure-responsive chemosensors, for example, mechanochromophores using thermally activated delayed fluorescence (TADF)^53^ and dynamic polymers using excimer^54^ and aggregation-induced emission (AIE).^55^ These discoveries prompted us to further explore such ratiometric chemosensors using novel operative mechanisms.

**Figure 1:**
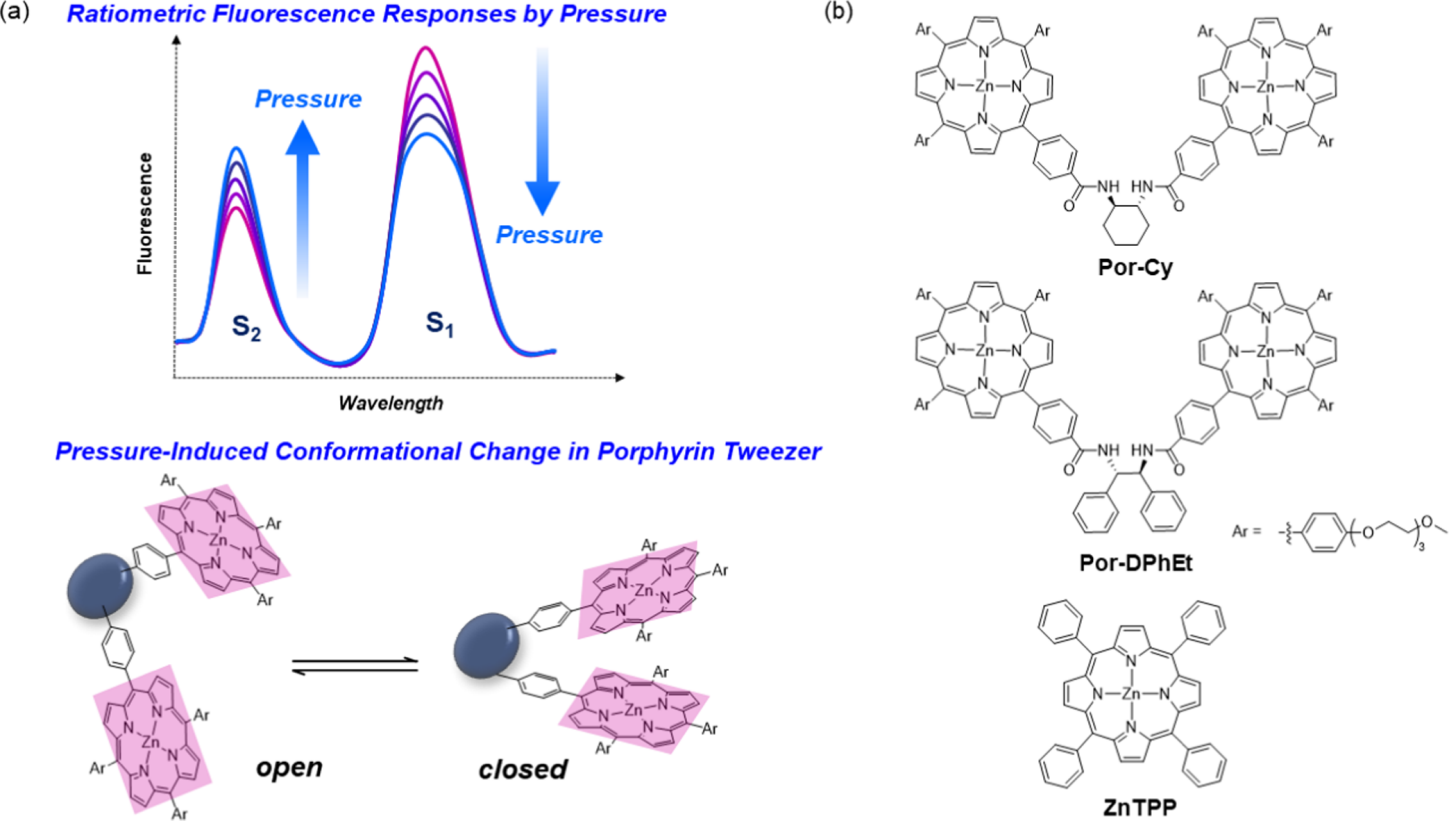
(a) Schematic illustration of the present concept of ratiometric fluorescence signaling using S_1_ and S_2_ states (top) and open-closed conformational change in porphyrin tweezer (bottom) induced by hydrostatic pressure. (b) Chemical structures of the porphyrin tweezers (**Por-Cy** and **Por-DPhEt**) and the reference compound **ZnTPP**.

In this study, we focused on S_2_ fluorescence, the signaling of which is still unexplored under hydrostatic pressures. A previous study on azulenes emitting S_2_ fluorescence was performed in polymer matrixes under high pressures (∼130 kbar or GPa order) and thus can be regarded as high-pressure solid chemistry.^56^ As shown in Figure 1a (top), ratiometric signaling, that is, using the S_1_/S_2_ ratio, may provide opportunities to fabricate ratiometric solution-state chemosensors. Among the many emissive organic molecules, fluorophores that emit S_2_ fluorescence are limited; however, porphyrin chromophores appear to be the best candidates because the S_1_ and S_2_ emissions are equally observed rather than azulenes or other S_2_ emitters.^57−59^ Therefore, as shown in Figure 1a (bottom), we hypothesized that every porphyrin unit connected by a flexible linker should interact with each other under hydrostatic pressurization. Berova et al. reported that porphyrin tweezers with different scaffolds can trap shorter and longer diamines at atmospheric pressure (0.1 MPa).^60,61^ This fact strongly reinforces our working hypothesis that such porphyrin tweezers dynamically change in response to hydrostatic pressure, that is, open-closed equilibrium. Herein, we report the results of our investigation on hydrostatic pressure-responsive porphyrin tweezers, **Por-Cy** and **Por-DPhEt** (Figure 1b); the ratiometric signaling of **Por-Cy** and **Por-DPhEt** is based on a new operative mechanism. The factors governing hydrostatic pressure ratiometry are elucidated based on the results and may enable the development of further smart chemosensors.

## Methods

### Materials

All commercial reagents and solvents were used as received without further purification. Fluorescence-free grade toluene was used as received for spectroscopy. The reference compound (**ZnTPP**) was synthesized according to a literature procedure.^62^

### Instruments

Melting points were measured using a Büchi apparatus. HR-MS spectra were obtained using a Bruker ESI micrOTOF II. ^1^H NMR spectra (400 MHz) were recorded using an ECX-400 spectrometer. ^1^H (500 MHz) and ^13^C NMR (125 MHz) spectra were recorded using an ECX-500 spectrometer. Ultraviolet/visible (UV/vis) spectra were measured in a quartz cell using a JASCO V-650 spectrometer equipped with a temperature controller. Fluorescence spectra were measured in a quartz cell using a JASCO FP-8500 instrument equipped with a temperature controller. Fluorescence lifetime decay was measured using a Hamamatsu Quantaurus-Tau single-photon counting apparatus fitted with an LED light source (λ_ex_ 405 nm). Circular dichroism (CD) spectra were recorded on a JASCO J-720WI instrument equipped with a temperature controller. Circularly polarized luminescence (CPL) spectra were recorded using a JASCO CPL-300 spectrofluoropolarimeter at room temperature (scattering angle: 0°).

### Hydrostatic Pressure Spectroscopy

The hydrostatic pressure experiments were performed according to an established procedure using a custom-built high-pressure apparatus (the photos are shown in the Supporting Information (SI)).^26,53,63^ Briefly, a quartz inner cell (light path of 2 mm) linked to a short Teflon tube was filled with a toluene solution of the sample, and the cell was set into the outer cell, where sapphire (other than CD) and YAG (for CD) windows were fitted. The closed outer cell was placed in the spectroscopic apparatus, and the hydrostatic pressurization ranged from 0.1 to ca. 400 MPa.

### General Synthetic Method

The desired porphyrin tweezers, **Por-Cy** and **Por-DPhEt**, were synthesized by condensing a Zn-coordinated porphyrin derivative comprising a monocarboxylic acid^64^ and each corresponding chiral diamino-scaffold; the detailed synthesis and characterization are described in the SI.

## Results and Discussion

### Scaffold Flexibility of Porphyrin Tweezer Chemosensors

**Figure 2:**
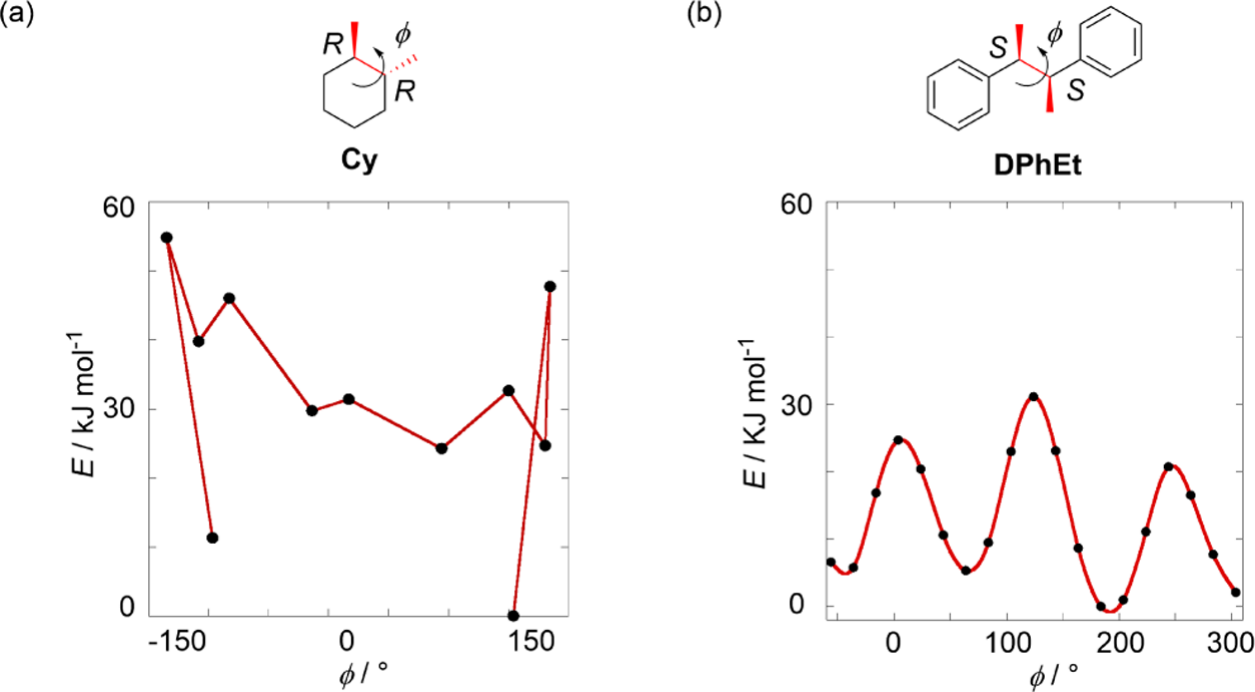
Potential energy landscape in dihedral angle changes of (a) **Cy** (data extracted from ref (64) were used) and (b) **DPhEt** calculated at ωB97xd/6-31G(d,p); the most stable conformers in each scaffold were set at 0 kJ/mol.

The open-closed performance that occurs in response to hydrostatic pressure is most probably responsible for scaffold flexibility in the tweezers. To determine the flexibility, that is, the rotational barrier, quantum chemistry calculations using density functional theory (DFT) were applied to the model skeletons (**Cy** and **DPhEt**); the energy–dihedral angle diagram was obtained, as shown in Figure 2. Predictably, the alkane chain in **DPhEt** can rotate at a lower energy (31 KJ) than the cyclohexane ring in **Cy**, which has a higher energy (55 KJ) owing to the half-chair transition.^65^ Thus, **Por-DPhEt** may be more susceptible to hydrostatic pressure stimulus compared with **Por-Cy**.

### Optical Properties under Atmospheric Pressure

**Figure 3:**
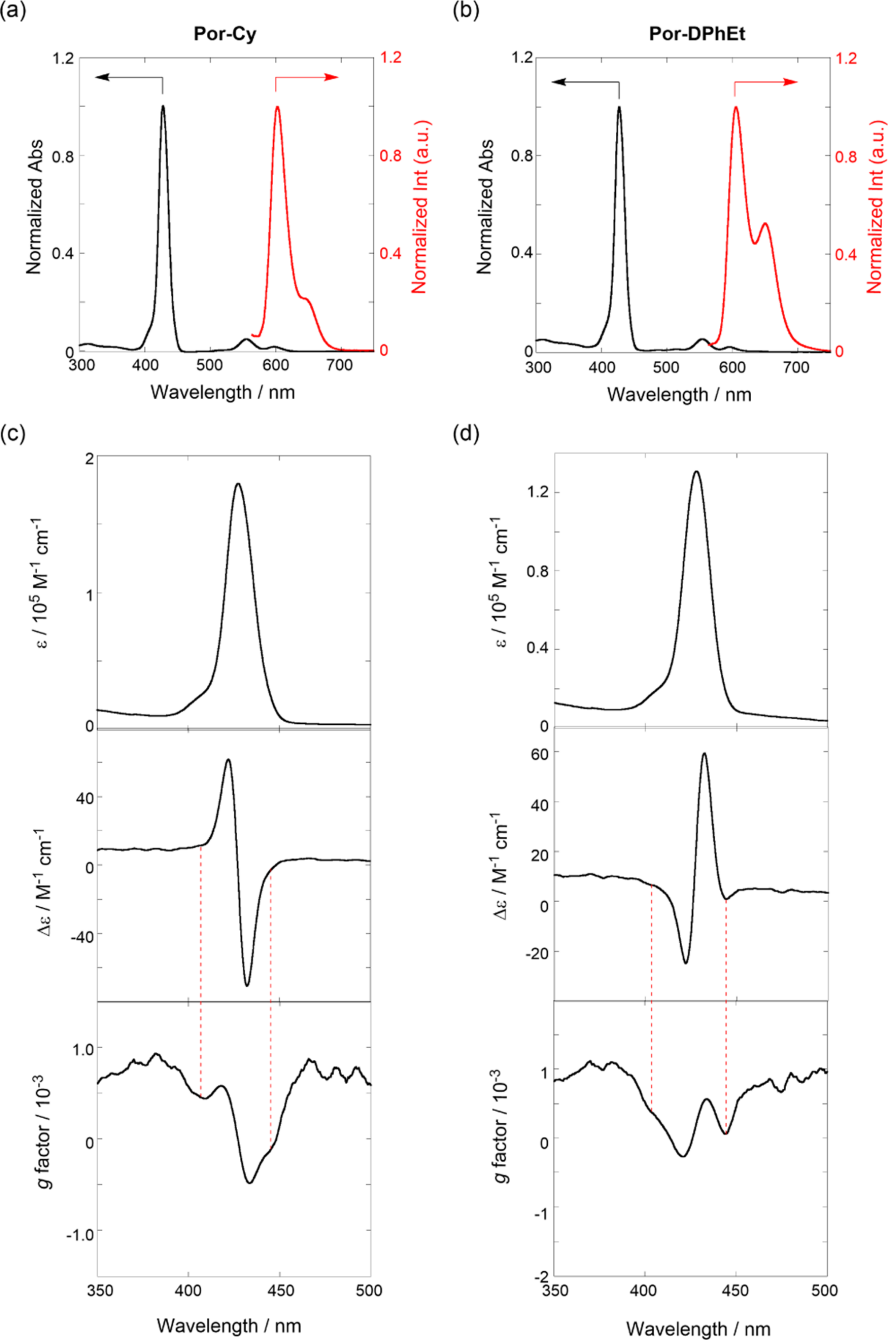
(a, b) Normalized UV/vis and fluorescence (λ_ex_ 554 nm) spectra in the whole wavelength region and (c,d) UV/vis (top), CD (middle), and *g* factor (bottom) spectra in the Soret region: (left) **Por-Cy** and (right) **Por-DPhEt** in toluene at 25 °C in a 1 cm cell.

First, we investigated the (chir)optical properties of the newly synthesized porphyrin tweezers **Por-Cy** and **Por-DPhEt** in toluene at 0.1 MPa. As shown in Figure 3a,b, both tweezers displayed Soret bands at 427 nm and Q bands at 555 and 597 nm; additionally, S_1_ fluorescence bands were observed at approximately 550–750 nm, and behaved similarly to those of regular zinc-coordinated tetraphenyl porphyrins.^60,66^ In the CD spectra and anisotropy (*g*) factor profile (Figure 3c,d), **Por-Cy** exhibited a strong bisignate Cotton effect with an amplitude of 132 M^–1^ cm^–1^. According to the exciton chirality theory,^67^ porphyrin chromophores are aligned in a counterclockwise manner. The chiral handedness originates from the chirality of the (*R*,*R*)-cyclohexane scaffold, the CD sign of which is consistent with that reported previously.^60^ By contrast, **Por-DPhEt** displayed a positive chirality in exciton coupling with an 84 M^–1^ cm^–1^ amplitude, indicating a clockwise alignment of the two porphyrins. This is also based on the (*S*,*S*)-scaffold chirality.^60^ The other minor CD bands, indicated by the red dashed lines, are discussed later.

**Figure 4:**
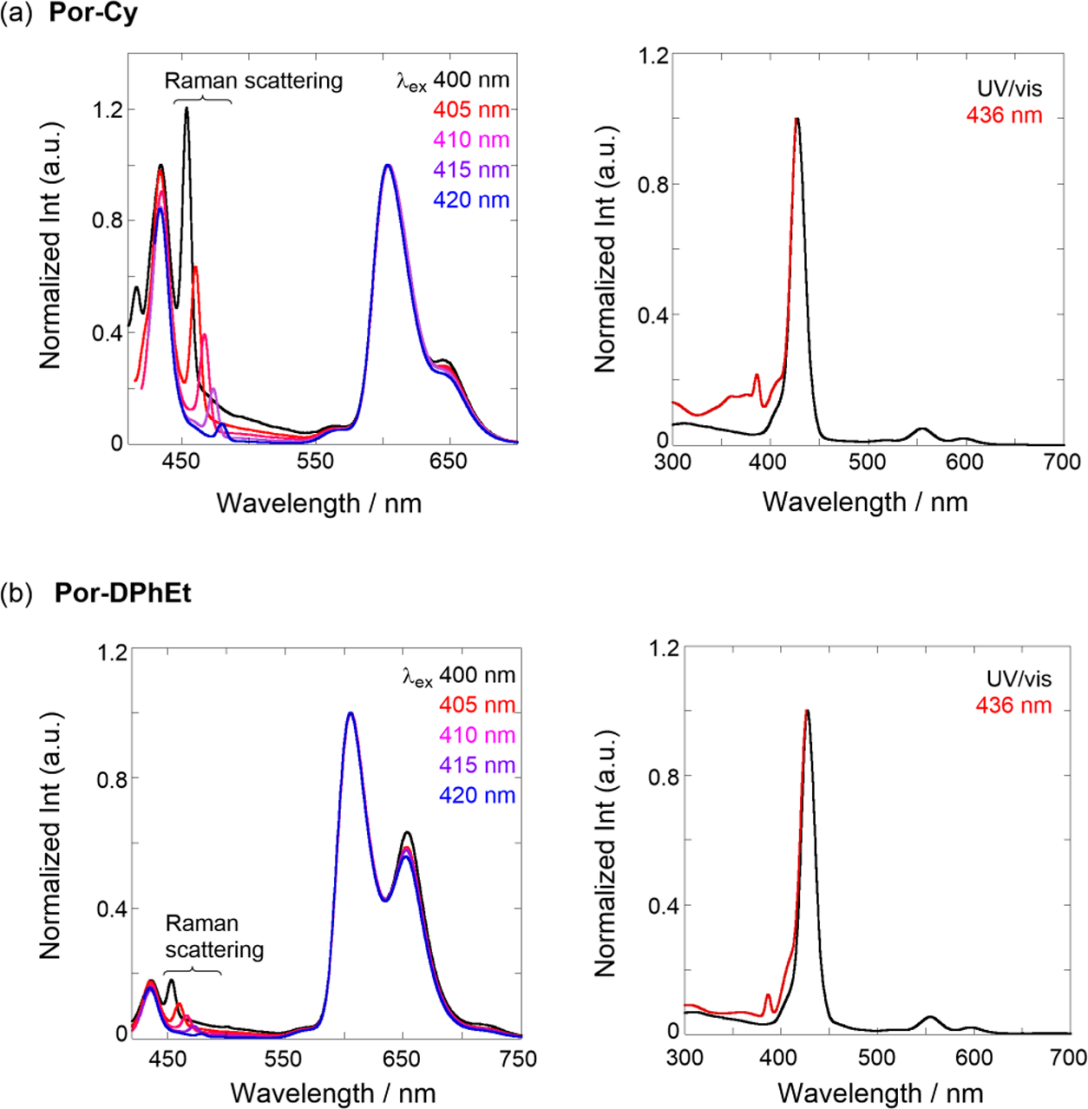
Normalized fluorescence (λ_ex_ 400–420 nm) (left) and excitation (λ_obs_ 436 nm) (right) spectra of (a) **Por-Cy** and (b) **Por-DPhEt** in toluene at 25 °C in a 1 cm cell.

Next, we investigated whether the two chemosensors emitted S_2_ fluorescence. As shown in Figure 4a (left), at excitation wavelengths of 400–420 nm in **Por-Cy**, regular fluorescence at 550–700 nm was observed at longer wavelengths than that of the Q bands, based on the Kasha rule, which was simply assignable to S_1_ fluorescence (vide supra). Interestingly, the fluorescence maxima (434 nm) located around the Soret band were observed following the anti-Kasha rule and were superimposable despite varying excitation; thus, this band could be determined as S_2_ fluorescence. Certainly, in Figure 4a (right), the excitation spectrum monitored at 436 nm in this fluorescence band matched the UV/vis absorption, particularly that of the Soret band; this fluorescence maximum is also similar to the S_2_ fluorescence of a previously reported Zn-coordinated porphyrin derivative.^57^ Therefore, the gradually changing peaks with varying excitations were simply the origin of Raman scattering. Similarly, in Figure 4b, the S_1_ and S_2_ fluorescence emissions at 550–750 and 435 nm originating from **Por-DPhEt** were determined from excitation wavelength dependency and excitation spectra. Furthermore, as listed in Table 1 (vide infra), the fluorescence decay profiles monitored at 436 nm had very short-lived excited species (<0.1 ns), beyond the detection limit of the ns-lifetime apparatus used in this study; however, the lifetimes of the short-lived components were consistent with previously reported values of 0.8–4.5 ps.^66^ These findings regarding the S_2_ emissions in the two chemosensors suggested that the porphyrin tweezers may enable the output of a ratiometric response based on the S_1_/S_2_ ratio upon hydrostatic pressurization. Thus, a conformational change, that is, open-closed equilibrium, in response to a hydrostatic pressure stimulus plays an important role in ratiometric signaling.

**Table 1:** Fluorescence Lifetimes of Tweezers in Toluene upon Hydrostatic Pressurization^a^

sensor	*P*/MPa	λ_obs_^b^/nm	*n*^c^	τ_1_/ns	*A*_1_	τ_2_/ns	*A*_2_	χ^2^
**Por-Cy**	0.1	436	2	<0.1	0.71	1.4	0.29	1.2
		605	1	1.5	1.0			1.3
	50	605	2	1.6	0.97	0.3	0.03	1.2
	100	605	2	1.5	0.97	0.2	0.03	1.1
	150	605	2	1.5	0.96	0.2	0.04	1.2
	200	605	2	1.5	0.96	0.3	0.04	1.3
	250	605	2	1.5	0.96	0.2	0.04	1.2
	300	605	2	1.5	0.95	0.3	0.05	1.2
**Por-DPhEt**	0.1	436	2	<0.1	0.62	1.3	0.38	1.1
		605	1	1.6	1.0			1.4
	50	605	2	1.6	0.98	0.2	0.02	1.1
	100	605	2	1.5	0.98	0.2	0.02	1.1
	150	605	2	1.5	0.98	0.3	0.02	1.5
	200	605	2	1.5	0.97	0.2	0.03	1.1
	250	605	2	1.5	0.97	0.2	0.03	1.1
	300	605	2	1.5	0.97	0.2	0.03	1.1

a Fluorescence lifetime (τ_i_/ns) and relative abundance (*A*_i_) of each excited species in non-degassed toluene at room temperature in a high-pressure cell; [**Por-Cy**] = 12.1 μM, [**Por-DPhEt**] = 9.7 μM.

b Monitoring wavelength.

c Number of components.

### Optical Properties and Ratiometric Signaling under Hydrostatic Pressure

To link the origins of the spectral changes with hydrostatic pressurization, we first performed concentration-dependent UV/vis spectral examinations at atmospheric (0.1 MPa) and high (300 MPa) pressures, the latter of which was the highest examined in this study. As shown in Figures S4 and S5 in the SI, for both tweezers, the Soret band maxima were straight lines as a function of the concentration ranges tested. Therefore, the following pressure-dependent spectral changes in the tweezers, at the concentration ranges within the calibration curves, originate from intramolecular conformational changes, that is, open-closed equilibration.

**Figure 5:**
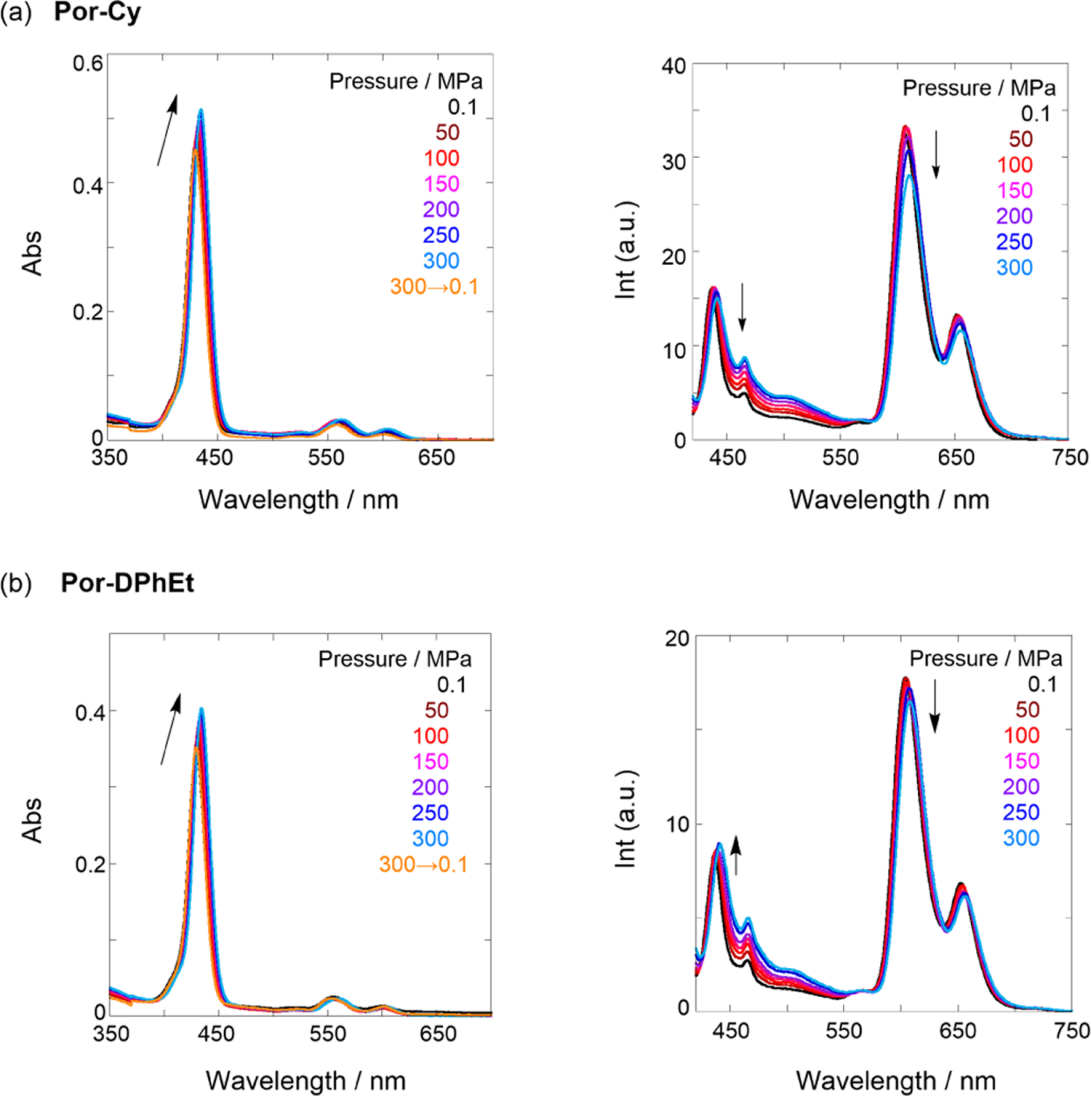
UV/vis (left) and fluorescence (λ_ex_ 410 nm) (right) spectra of (a) **Por-Cy** (12.1 μM) and (b) **Por-DPhEt** (9.7 μM) in toluene at 0.1, 50, 100, 150, 200, 250, and 300 MPa (from black to sky blue lines) at room temperature in a high-pressure cell. The orange line represents UV/vis spectra at 0.1 MPa, depressurized from 300 MPa.

Next, we investigated the hydrostatic pressure responses of the two tweezers using UV/vis absorption and fluorescence spectroscopy. As shown in Figure 5a,b (left), the UV/vis spectra at 0.1 MPa (orange lines) depressurized from those at 300 MPa (sky blue lines) and were superimposable on the original spectra (black lines), indicating that pressurization is a reversible process. The hydrostatically pressurized UV/vis absorption at the Soret bands showed steady bathochromic shifts of −0.917 cm^–1^ MPa^–1^ for **Por-Cy** and −0.861 cm^–1^ MPa^–1^ for **Por-DPhEt** (Figure S6 in the SI) and hyperchromic effects with increasing hydrostatic pressure. The former behavior arises from the medium (solvent) polarizability (density) changes induced by hydrostatic pressurization, causing the stabilization of π* orbital via solvation;^35^ these shifts appear to be similar to those obtained from the π systems we previously reported.^26^ The latter occurs simply because of the increasing effective concentration based on pressurization-induced solvent compression. The hydrostatic pressure-fluorescence spectra of the two tweezers displayed contrasting behaviors, as shown in Figure 5a,b (right). Namely, **Por-Cy** exhibited a gradual decrease in intensity for both S_1_ and S_2_; by contrast, **Por-DPhEt** displayed a similar decrease for S_1_ yet a steady increase for S_2_. Nevertheless, the two tweezers displayed isoemissive points at 578 nm for **Por-Cy** and 571 nm for **Por-DPhEt**, respectively, indicating the existence of two conformers, i.e., open-closed equilibrium. This contrasting behavior is attributed to the differently stacked (closed) species of the two tweezers. Hence, we should separately discuss the proof of (1) the existence of an open-closed equilibrium and (2) the contrasting responses of S_2_ intensity to hydrostatic pressurization.

### Open-Closed Equilibration in the Tweezers under Hydrostatic Pressures

At an atmospheric condition of 0.1 MPa, the CD spectra and anisotropy (*g*) factor profiles shown in Figure 3c,d (red dashed lines) provide strong evidence. In **Por-Cy**, the maximum at 409 nm and negative shoulder at 448 nm, other than that of regular exciton coupling (open species), can be reasonably assigned to an H-type closed species for the former and J-type for the latter, respectively, according to Kasha’s exciton theory.^68^ Similarly, in **Por-DPhEt**, the shorter-wavelength shoulder at 400 nm and distinctive maximum at the longer wavelength of 444 nm, other than that of open species-based exciton coupling, were also ascribed to H- and J-type closed species. These results support the existence of an open-closed (J and H mixtures) equilibration at 0.1 MPa. The following hydrostatic pressurization measurements displayed an interesting equilibration trend. As listed in Table 1, in **Por-Cy**, the fluorescence lifetime decay at 0.1 MPa was fitted to a monoexponential function to afford τ_1_ as 1.5 ns, which is reasonably assignable to the open conformer as the major species (see all decay profiles in Figures S7 and S8 in the SI). The closed mixtures might have been present in too low quantities to be detected as emissive species. By contrast, the lifetime decay profiles upon hydrostatic pressurization were reasonably fitted to a sum of two exponential functions to afford τ_1_ and τ_2_ as 0.2–0.3 ns, the latter of which was assignable to the closed species owing to the promotion of stacking-based radiationless deactivation.^69^ This result is consistent with S_1_ fluorescence quenching behavior upon hydrostatic pressurization (Figure 5a, right).

**Figure 6:**
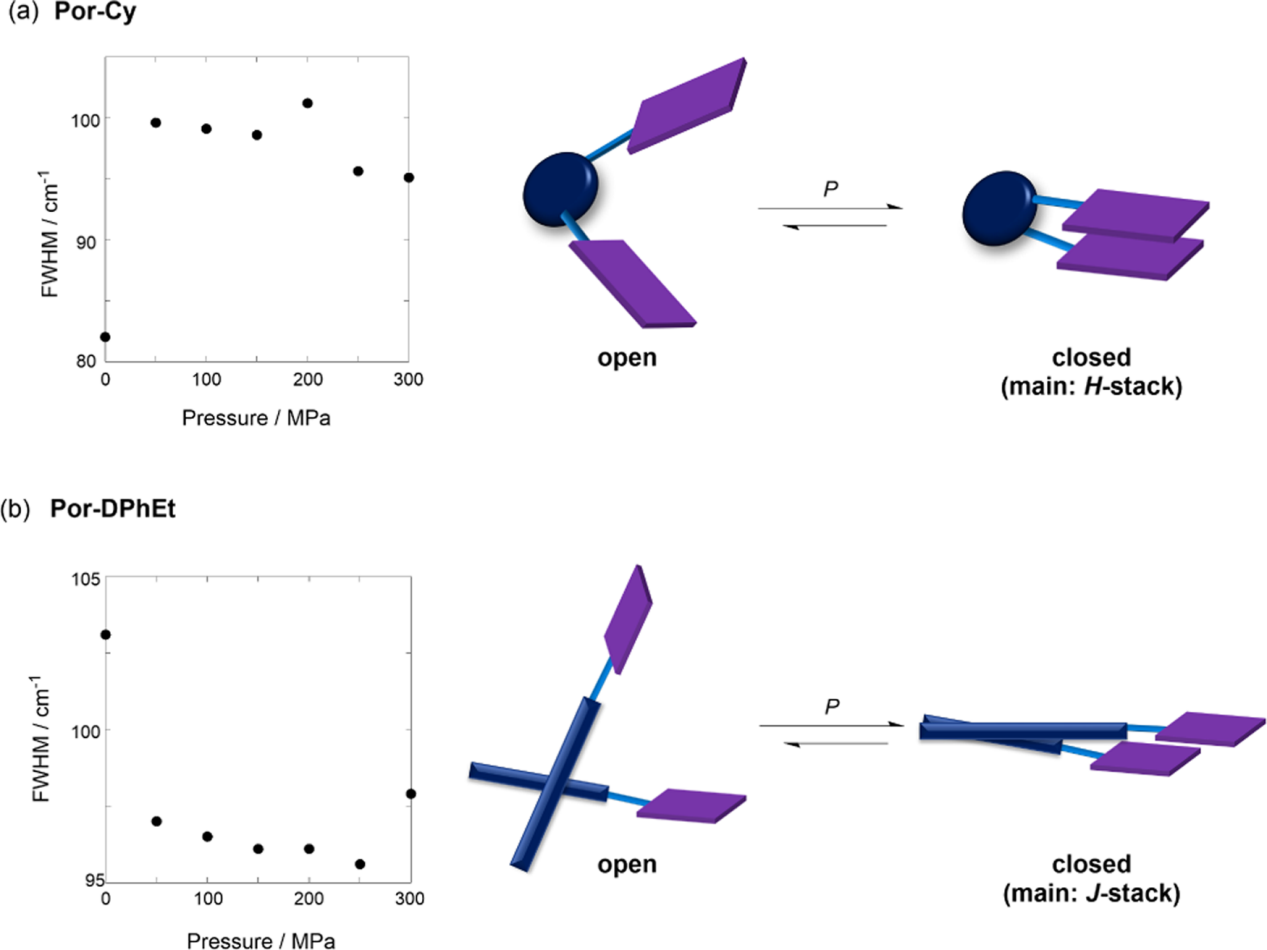
Hydrostatic pressure-dependent full width at half-maximum (fwhm) in the Soret band (left) and open-closed conformational changes (right) of (a) **Por-Cy** and (b) **Por-DPhEt**.

Further important aspects were revealed by the analysis of the hydrostatic pressure-dependent UV/vis spectral changes. The changes in the full width at half-maximum (fwhm) at the Soret band at approximately 390–460 nm were investigated owing to the overlap with all of the species as open and closed conformers. As shown in Figure 6a (left), the fwhm was widened upon hydrostatic pressurization, indicating that a new band developed at the shorter wavelength in association with the pressure-induced bathochromic shifts; the H-type closed conformer develops upon hydrostatic pressurization. Hence, the CD/*g* spectra, hydrostatic pressure lifetime decays, steady-state fluorescence, and fwhm of the UV/vis spectra support the idea that the original state is an open conformer with small amounts of H/J-stacked species equilibrated to the H-type closed state upon hydrostatic pressurization, as illustrated in Figure 6a (right).

By contrast, **Por-DPhEt** exhibited opposite stacking behavior under pressure; the lifetime of τ_2_ = 0.2–0.3 ns (Table 1) and the S_1_ fluorescence quenching (Figure 5b, right) were similar to those for **Por-Cy**. Interestingly, the pressurized fwhm changes exhibited an opposite narrowing behavior, as shown in Figure 6b (left). This result can be easily explained in terms of a new band developing at longer wavelengths by canceling out the effects of pressure-induced bathochromic shifts; thus indicating the formation of a *J*-stacked conformer, as shown in Figure 6b (right). This contrasting effect of pressure on stacking formation is probably responsible for the structural difference of the scaffold, which is easily controlled by hydrostatic pressure.

### S_2_ Fluorescence Responses under Hydrostatic Pressure

**Figure 7:**
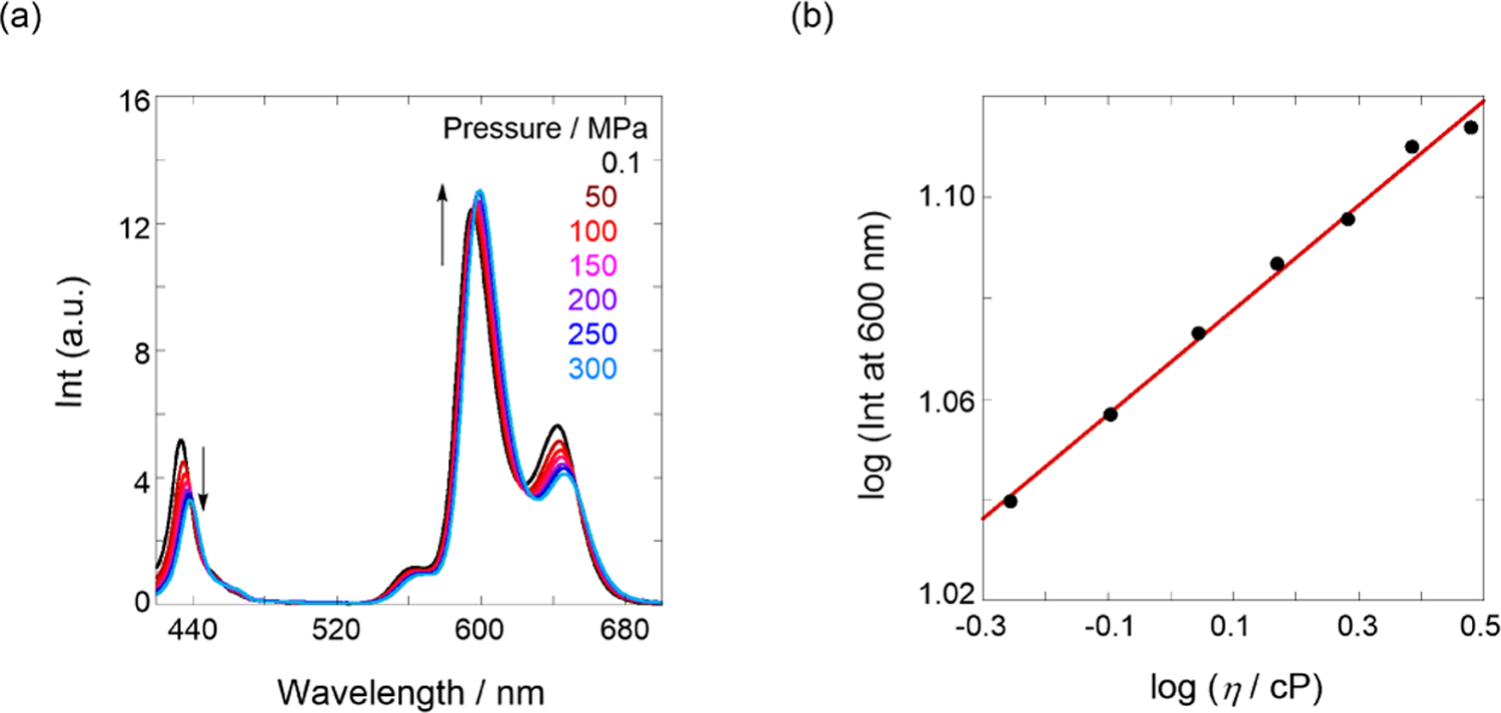
(a) Fluorescence (λ_ex_ 410 nm) spectra of **ZnTPP** (2.9 μM) in toluene at 0.1, 50, 100, 150, 200, 250, and 300 MPa (from black to sky blue lines) at room temperature in a high-pressure cell. (b) Logarithmic plot of fluorescence intensities at 600 nm obtained in (a) against viscosity (η) changes, according to the Förster–Hoffmann equation; pressure-dependent η of toluene was estimated by the data in ref (71)

To understand the factors that govern the S_2_ responses during hydrostatic pressurization, we examined the hydrostatic pressure-fluorescence spectra of the corresponding reference compound, **ZnTPP** (see structure in Figure 1b). As shown in Figure 7a, the S_1_ fluorescence intensity increases, whereas that of S_2_ decreases with increasing hydrostatic pressure. The pressure-dependent S_1_ behavior can be easily accounted for by the fact that S_1_ fluorescence increased due to the inhibition of collisional deactivation by solvent attack based on the pressure-induced increase in the viscosity of the solvent. This phenomenon is well known in general π-systems;^50,53^ according to the Förster–Hoffmann equation (log(Int) = *B* log(η) + *C*);^70^ in Figure 7b, the slope was estimated by the plot and afforded *B* as 0.104. Surprisingly, the S_2_ fluorescence response was opposite to that of the S_1_ state.

At this stage, we do not know if the pressure-responsive decreasing S_2_ behavior is generalized in π-systems or restricted to porphyrin derivatives, as very few organic molecules emit S_2_ fluorescence. However, in the porphyrin derivative, we discovered that S_2_ fluorescence decreased in intensity with increasing hydrostatic pressure owing to the promotion of radiationless deactivation/inhibition of the fluorescence rate at a higher state level. This phenomenon may arise because the difference in the π-electron clouds in the S_1_ and S_2_ states^57^ is affected contrarily by pressure-induced solvent character changes, such as viscosity, polarity, and density.^35,72−74^ Nevertheless, this fact enabled us to explain the contrasting S_1_/S_2_ responses in the two tweezers. The *H*-stacked mode in **Por-Cy** exhibited the same trend as that of the reference **ZnTPP**, in contrast to the *J*-stacked mode in **Por-DPhEt**. This contrasting trend observed in the tweezers may originate from the different excited-state microenvironments around the two (H or J) stacked structures.

### Quantifying Hydrostatic Pressure by Using Two Tweezers

**Figure 8:**
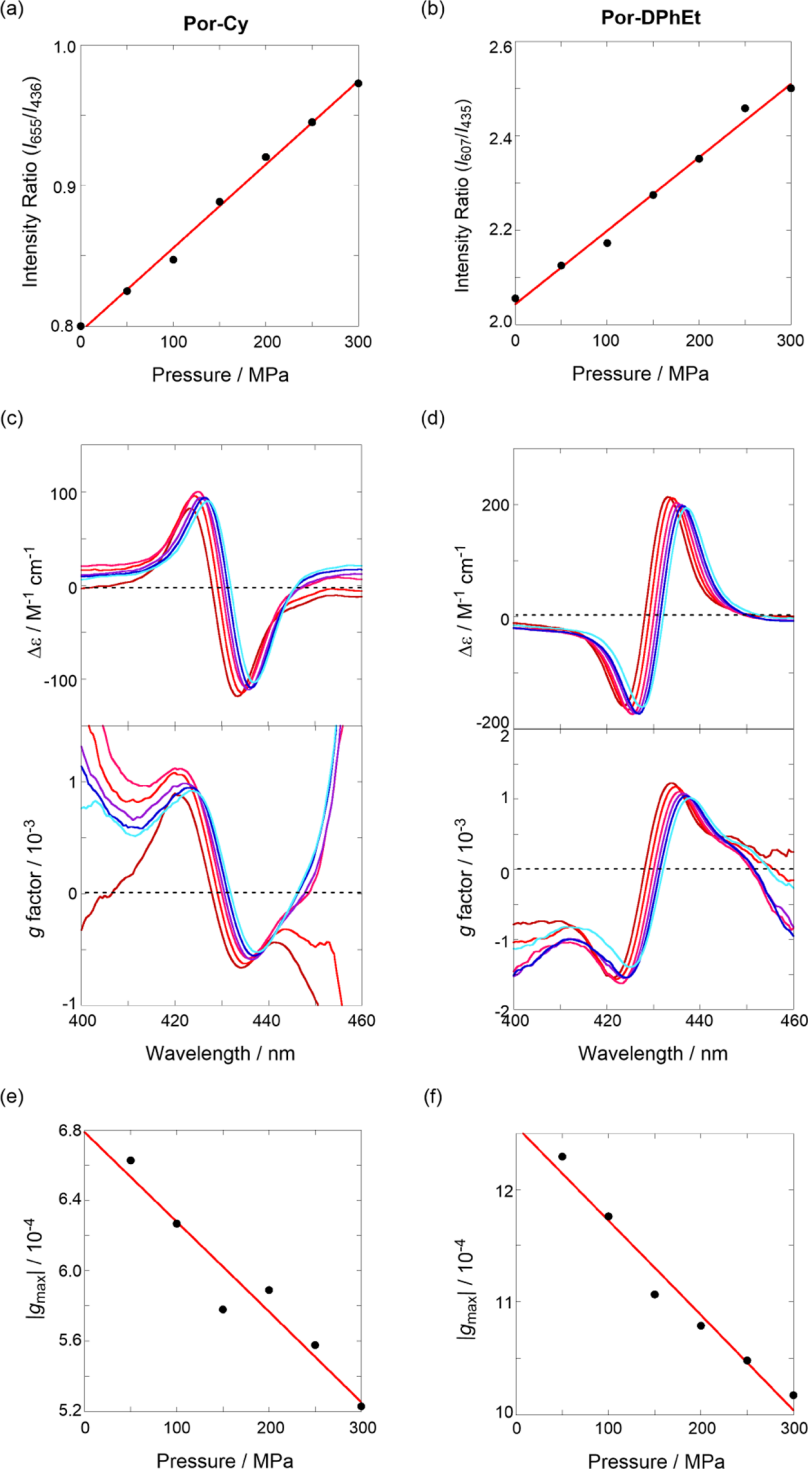
(Left) **Por-Cy** and (right) **Por-DPhEt**: (a, b) plots of fluorescence intensity ratios against pressure (correlation coefficient *r* = 0.997 (left) and 0.995 (right)), (c, d) pressure-dependent CD (top) and *g* factor (bottom) spectra in the Soret region in toluene at 50, 100, 150, 200, 250, and 300 MPa (from brown to sky blue lines), and (e, f) plots of |*g*_max_| in the first Cotton effects obtained in (c, d) against pressure *r* = 0.963 (left) and 0.983 (right).

As we revealed the factors controlling the fluorescence ratiometric signals that are distinctively different from the two tweezers based on pressure-induced stacking behaviors, we compared the ratiometry; the response factor γ was defined as the ratio of S_1_/S_2_ fluorescence intensity. The two tweezers displayed good straight lines (Figure 8a,b), thereby enabling the quantification of the hydrostatic pressure stimulus using two tweezer chemosensors. More importantly, the γ value of 1.6 × 10^–3^ MP^–1^ in **Por-DPhEt** is 2.7-fold larger than that of 5.9 × 10^–4^ MP^–1^ in **Por-Cy**. The higher responsiveness toward hydrostatic pressurization in **Por-DPhEt** is likely due to the flexible linker based on the energy barrier (see Figure 2 and the relevant discussion). Hydrostatic pressure-CD spectroscopy is an extremely powerful tool for obtaining deeper mechanistic insight into the linker structure. As shown in Figure 8c,d, the first Cotton effects at longer wavelengths in both tweezers gradually decreased with increasing hydrostatic pressure, and the *g*-based response factor γ_*g*_abs__ was estimated in the plot of |*g*_max_| against pressure (Figure 8e,f). The γ_*g*_abs__ value of −8.4 × 10^–7^ MP^–1^ in **Por-DPhEt** is also larger (1.6 times) than that of −5.1 × 10^–7^ MP^–1^ in **Por-Cy**, indicating that ground-state (chiral) conformational change induced by hydrostatic pressure is the origin of the different pressure responsiveness, that is, the flexibility of the scaffold.

**Figure 9:**
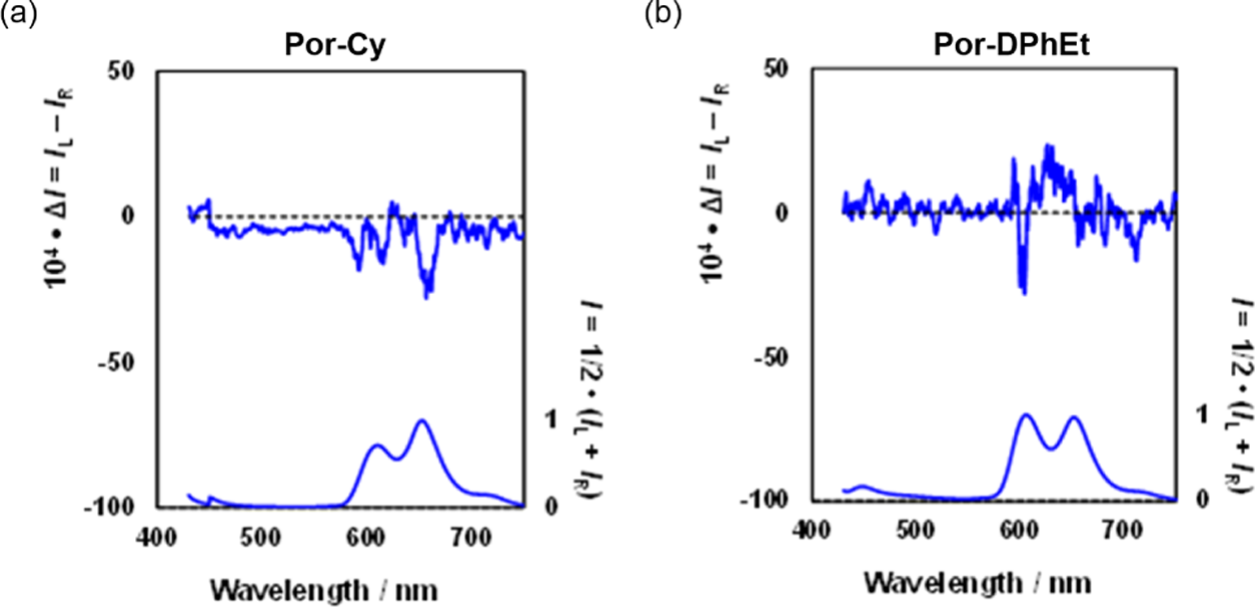
CPL (top) and PL (bottom) (λ_ex_ 400 nm) spectra of (a) **Por-Cy** (10 μM) and (b) **Por-DPhEt** (10 μM) in toluene at room temperature in a 1 cm cell.

We realized that these tweezers are chiral emitters and that the ratiometric character of CPL^75,76^ could also fulfill the present aim, that is, ratiometric signaling. *g*_lum_ is an anisotropy factor for CPL, and is expressed in eq 1 as follows
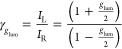
1where *I*_L_ and *I*_R_ represent left- and right-handed CPL, respectively. Thus, the ratio of *I*_L_/*I*_R_ defined as γ_*g*_lum__, is a chiral emission ratiometric factor. As shown in Figure 9, at 0.1 MPa, **Por-Cy** in the S_1_ region showed a distinctive negative CPL (|*g*_lum_| = 2.9 × 10^–3^) but was undetectable in S_2_. By contrast, **Por-DPhEt** exhibited negligible CPL peaks in both states, probably because of the flexible linker. The γ_*g*_lum__ value of **Por-Cy** in S_1_ was estimated to be 1.0029 according to eq 1. This value is comparable to those (1.0021–1.0032) obtained for pyrene-modified polylysine chemosensors,^54^ in which chiral pyrene excimers function as CPL emitters. Chiral ratiometry obtained from chirally twisted excitons in the tweezers should lead to a novel chiral index for quantifying the chiroptical properties of fluorescent materials.

## Conclusions

We discovered a pressure-responsive chemosensor, a porphyrin tweezer, with ratiometric signaling, in which a novel mechanism, that is, the S_1_/S_2_ fluorescence ratio, functions to quantify hydrostatic pressure stimuli. The ratiometry relating to the higher excited S_2_ state can be further controlled by the open-closed conformational change based on the inherent nature of the tweezer. Notably, linker flexibility in the tweezer skeleton significantly affects the degree of ratiometry. The tweezers discussed herein, particularly **Por-Cy**, also displayed a notable chiral ratiometric performance of γ_*g*_lum__ = 1.0029, which may lead to the development of a further chiral fluorescent chemosensor in response to hydrostatic pressure and, possibly, other stimuli. Hence, this study provides appropriate guidelines for fabricating smart chemosensors capable of quantifying hydrostatic pressure using ratiometric signaling.

## Supporting Information Available

Photos for the pressure apparatus; synthesis and characterization data; additional spectroscopic and decay data; and computational simulations (PDF)
