# Supplementary material for: Ratiometric Chemosensors That Are Capable of Quantifying Hydrostatic Pressure Stimulus: A Case of Porphyrin Tweezers
Source: ACS Phys Chem Au. 2024 Jul 12;4(5):510–21. doi: 10.1021/acsphyschemau.4c00025 (PMC11447962; doi:10.1021/acsphyschemau.4c00025)
Supplement: Supplementary file 1 — pg4c00025_si_001.pdf [file pg4c00025_si_001.pdf]

Supporting Information
for

Ratiometric Chemosensors That Are Capable of Quantifying Hydrostatic Pressure Stimulus: A Case of Porphyrin Tweezers

Seiya Ono,[†] Tomokazu Kinoshita,[†] Hiroshi Iwasaki,[‡] Yoshitane Imai,[‡] and Gaku Fukuhara[*,†]

[†] *Department of Chemistry, Tokyo Institute of Technology, 2-12-1 Ookayama, Meguro-ku, Tokyo 152-8551, Japan*

[‡] *Department of Applied Chemistry, Graduate School of Science and Engineering, Kindai University, 3-4-1 Kowakae, Higashi-Osaka, Osaka 577-8502, Japan*

E-mail: gaku@chem.titech.ac.jp

Hydrostatic Pressure Apparatus

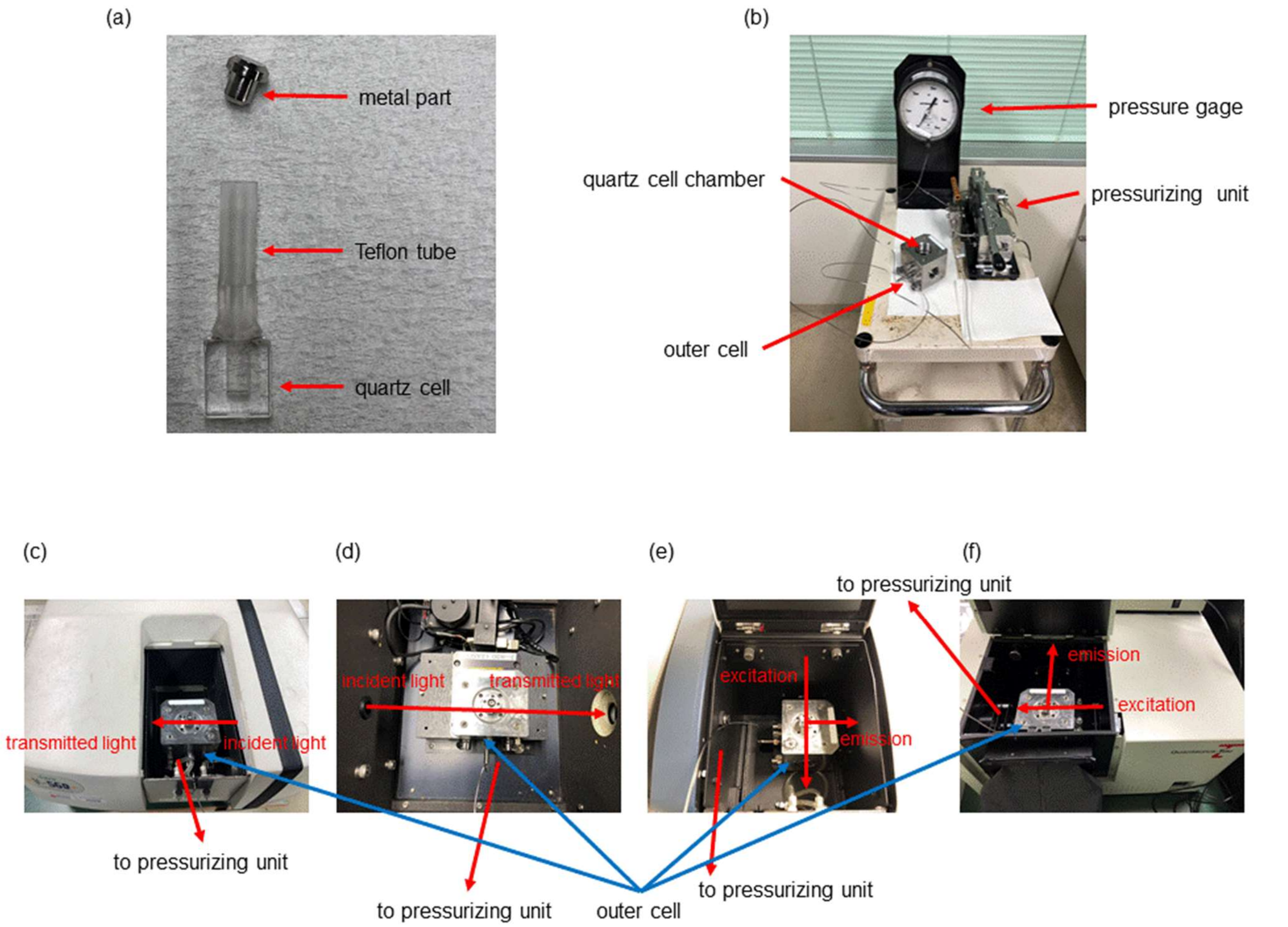

Figure S1. (a) Inner quartz cell, (b) pressurizing units, and setup for (c) UV/vis, (d) circular dichroism, (e) fluorescence, and (d) lifetime measurements. Reproduced with permission from ref 63. Copyright 2020 John Wiley & Sons.

Synthesis and Characterization

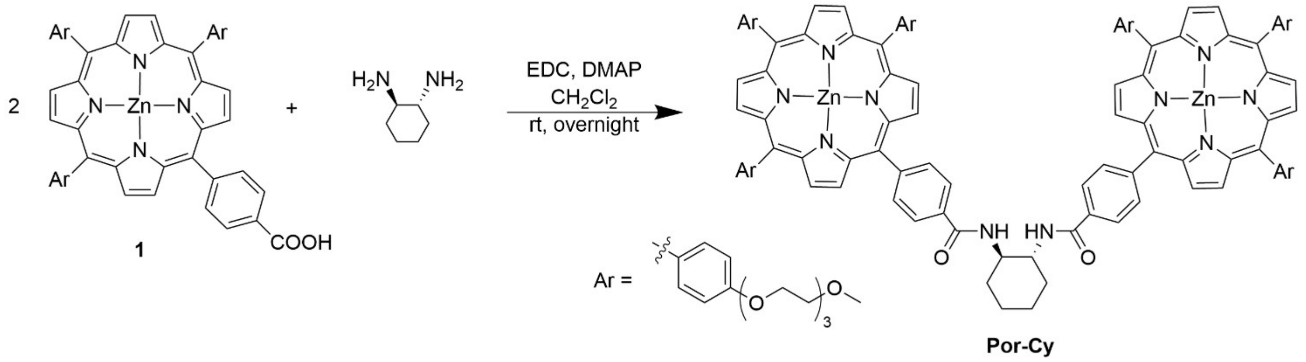

Por-Cy. 1-Ethyl-3-(3-dimethylaminopropyl)carbodiimide hydrochloride (EDC) (85.7 mg, 440 μmol) and *N*,*N*-dimethyl-4-aminopyridine (DMAP) (58.0 mg, 440 μmol) were added to a CH_2Cl_2 solution (10 mL) containing **1**[1] (55.5 mg, 44 μmol) and (1*R*,2*R*)-1,2-cyclohexanediamine (2.7 mg, 18 μmol) in a flask, and stirred for 18 h at room temperature. The reaction mixture was poured into water and extracted with CH_2Cl_2. The combined extracts were washed with brine and dried over anhydrous Na_2SO_4. The filtrated solvent was removed by evaporation. The residue was purified using aluminum column chromatography (CH_2Cl_2/MeOH = 200:1–100:1) to obtain **Por-Cy** as a blue solid (8.1 mg) in 18% yield; mp 87–90 °C; HR-MS (ESI, TOF) *m*/*z* $[M+2Na]^{2+}$ calcd for $C_{138}H_{150}N_{10}O_{26}Zn_2Na_2$ 1268.4545, found 1268.9560; ^{1}H NMR (400 MHz, $CDCl_3$) δ 8.86-8.77 (m, 24H), 8.20-8.09 (s, 8H), 7.91 (m, 8H), 6.91 (m, 6H), 4.60 (m, 2H), 4.41-3.20 (m, 90H), 2.30 (s, 2H), 1.90 (s, 2H), 1.45 (s,2H), 1.91 (s, 2H); ^{13}C NMR ($CDCl_3$, 125 MHz) δ_C 149.7, 135.5, 132.3, 131.1, 129.0, 112.6, 72.1, 70.5, 65.3, 59.2, 29.8, 28.7, 25.9, 14.1.

[1] Fukuhara, G.; Sasaki, M.; Numata, M.; Mori, T.; Inoue, Y. *Chem. Eur. J.* **2017**, *23*, 11272–11278.

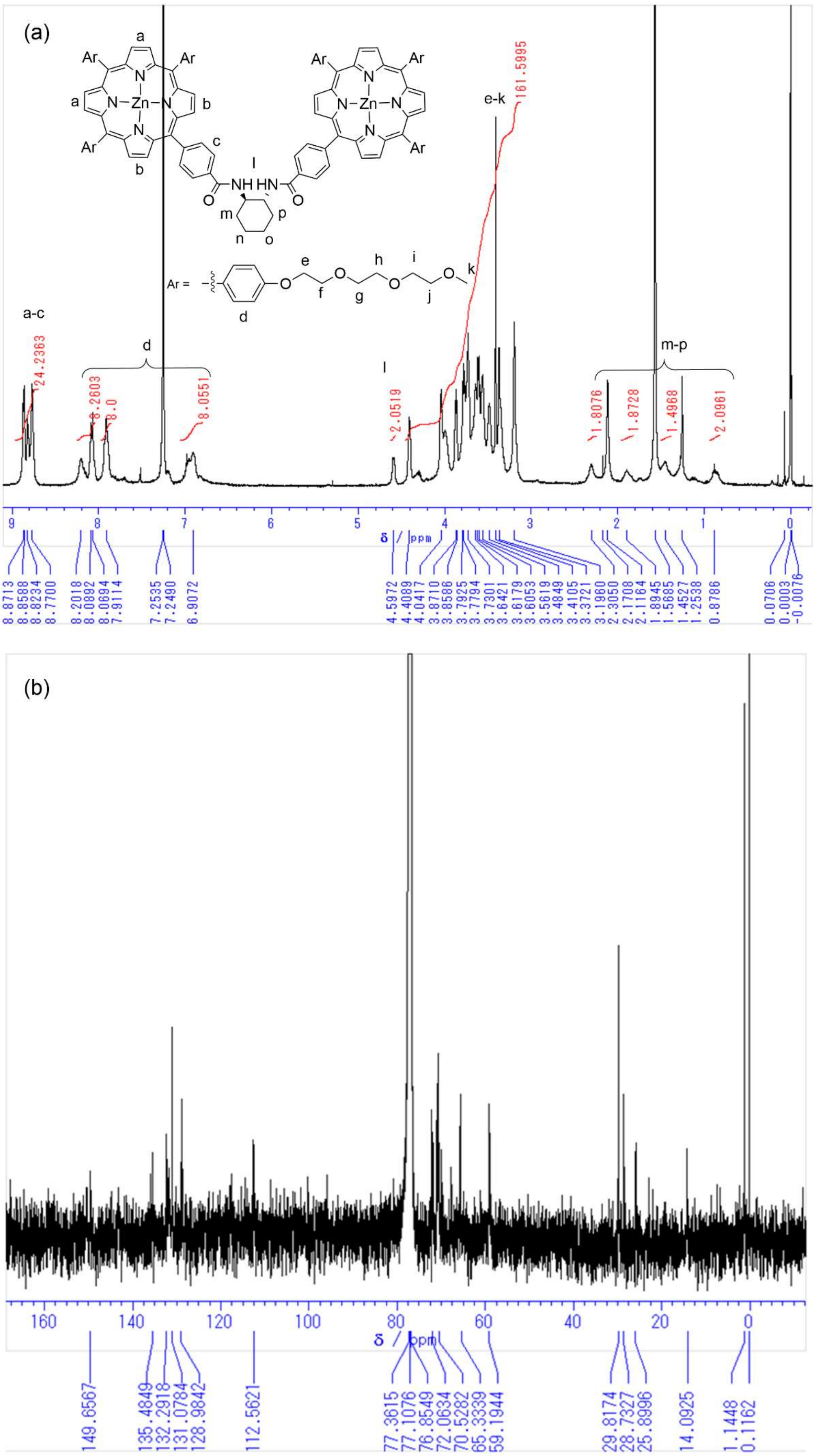

Figure S2. (a) ^{1}H NMR (400 MHz) and (b) ^{13}C NMR (125 MHz) spectra of **Por-Cy** in $CDCl_3$ at room temperature.

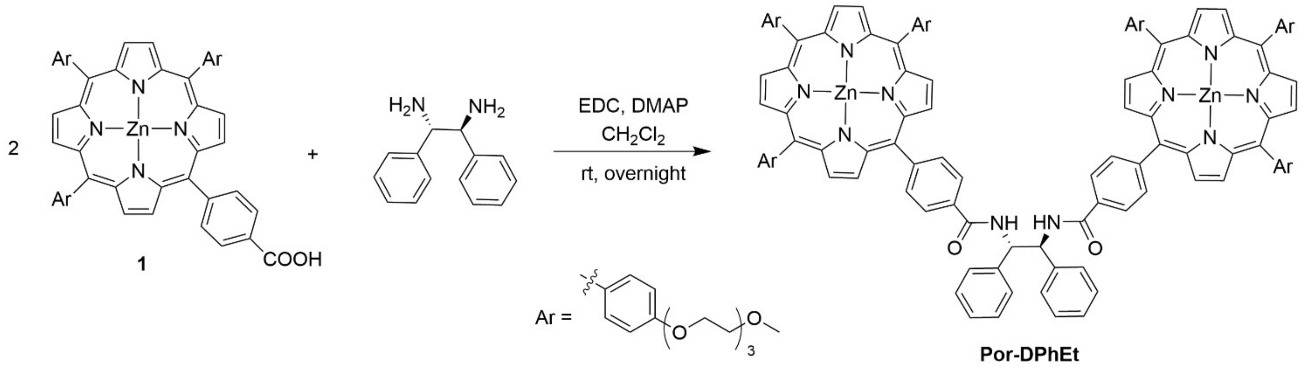

Por-DPhEt. EDC (40.0 mg, 209 μmol) and DMAP (40.0 mg, 327 μmol) were added to a CH_2Cl_2 solution (20 mL) of **1** (21.4 mg, 8.25 μmol) and (1*S*,2*S*)-(-)-1,2-diphenyl-1,2-ethanediamine (1.0 mg, 4.7 μmol) in a flask, and stirred for 18 h at room temperature. The reaction mixture was poured into water and extracted with CH_2Cl_2. The combined extracts were washed with brine and dried over anhydrous Na_2SO_4. The filtrated solvent was removed by evaporation. The residue was purified using aluminum column chromatography (CH_2Cl_2/MeOH = 180:1–175:1) to afford **Por-DPhEt** as a blue solid (10.3 mg) in 64% yield; mp 75–77 °C; HR-MS (ESI, TOF) *m*/*z* $[M+Na]^+$ calcd for $C_{146}H_{152}N_{10}O_{26}Zn_2Na$ 2611.9360, found 2611.9336; ^1H NMR (400 MHz, $CDCl_3$) δ 8.95-8.72 (m, 24H), 8.26-8.09 (s, 12H), 7.78-7.70 (m, 12H), 6.81-6.71 (m, 10H), 4.41-4.28 (m, 12H), 4.13 (s, 2H), 4.04-3.02 (m, 78H); ^{13}C NMR ($CDCl_3$, 125 MHz) δ_C 150.6, 149.7, 135.3, 131.8, 128.9, 112.7, 72.1, 71.1, 68.0, 65.6, 59.1, 53.4, 35.0, 28.6.

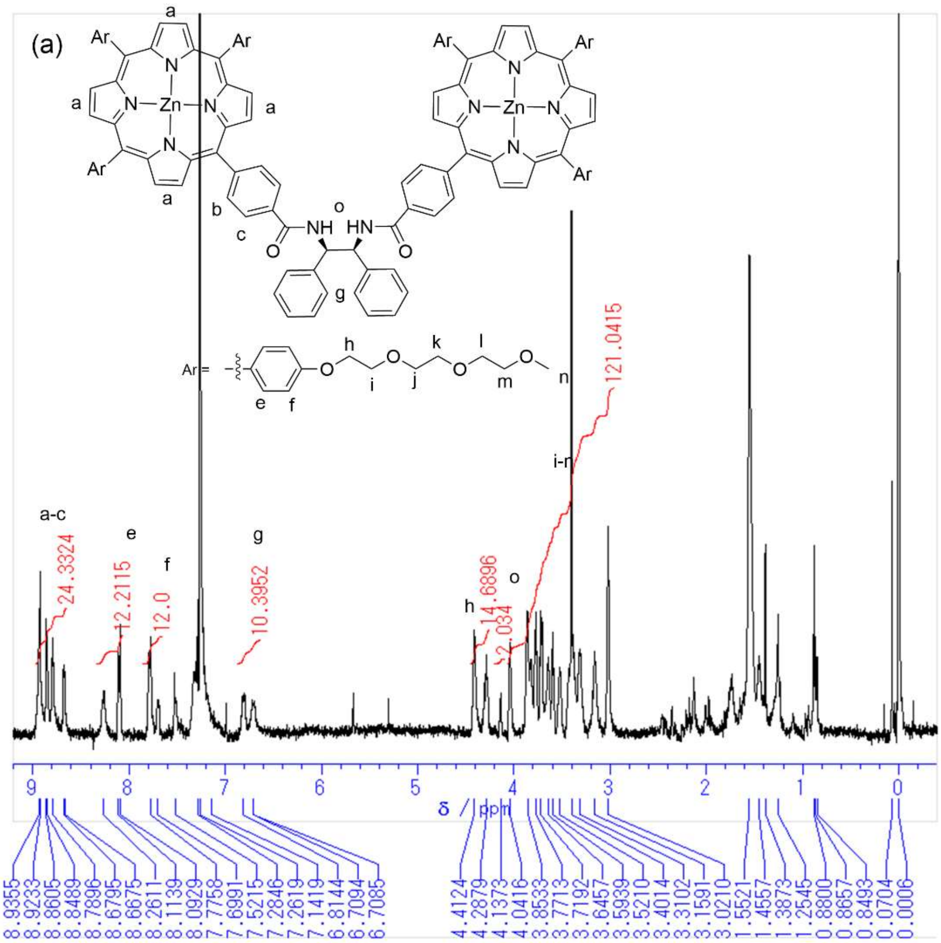

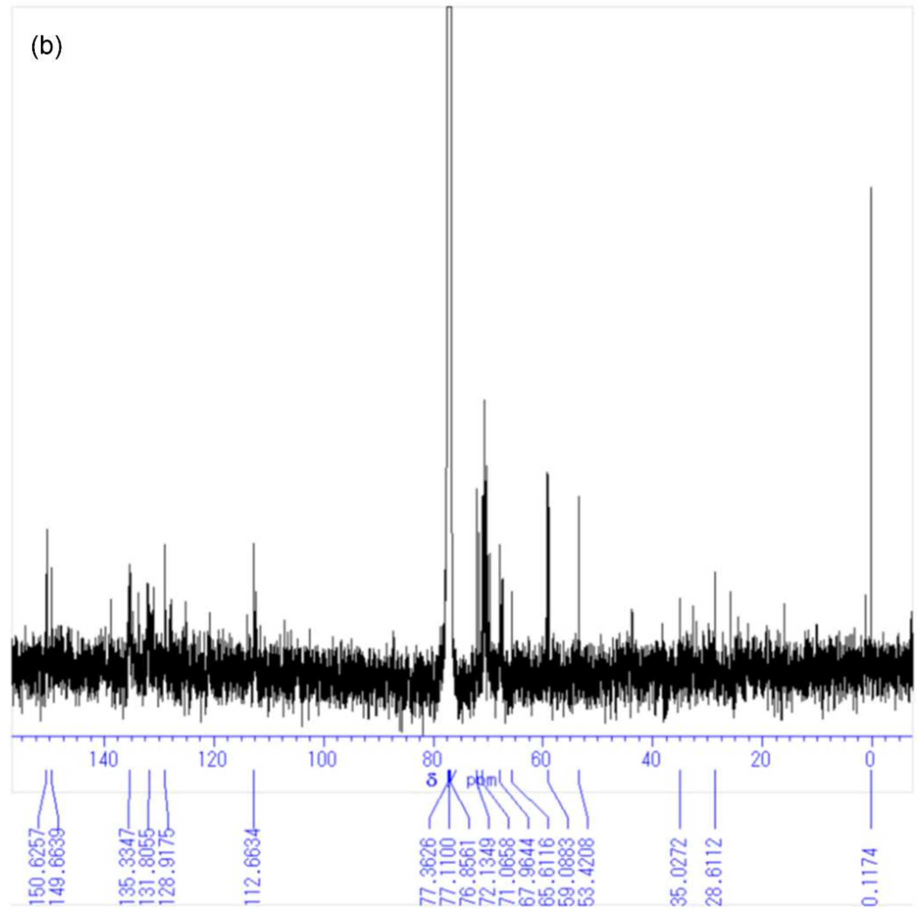

Figure S3. (a) ^{1}H NMR (400 MHz) and (b) ^{13}C NMR (125 MHz) spectra of **Por-DPhEt** in $CDCl_3$ at room temperature.

Concentration Dependencies

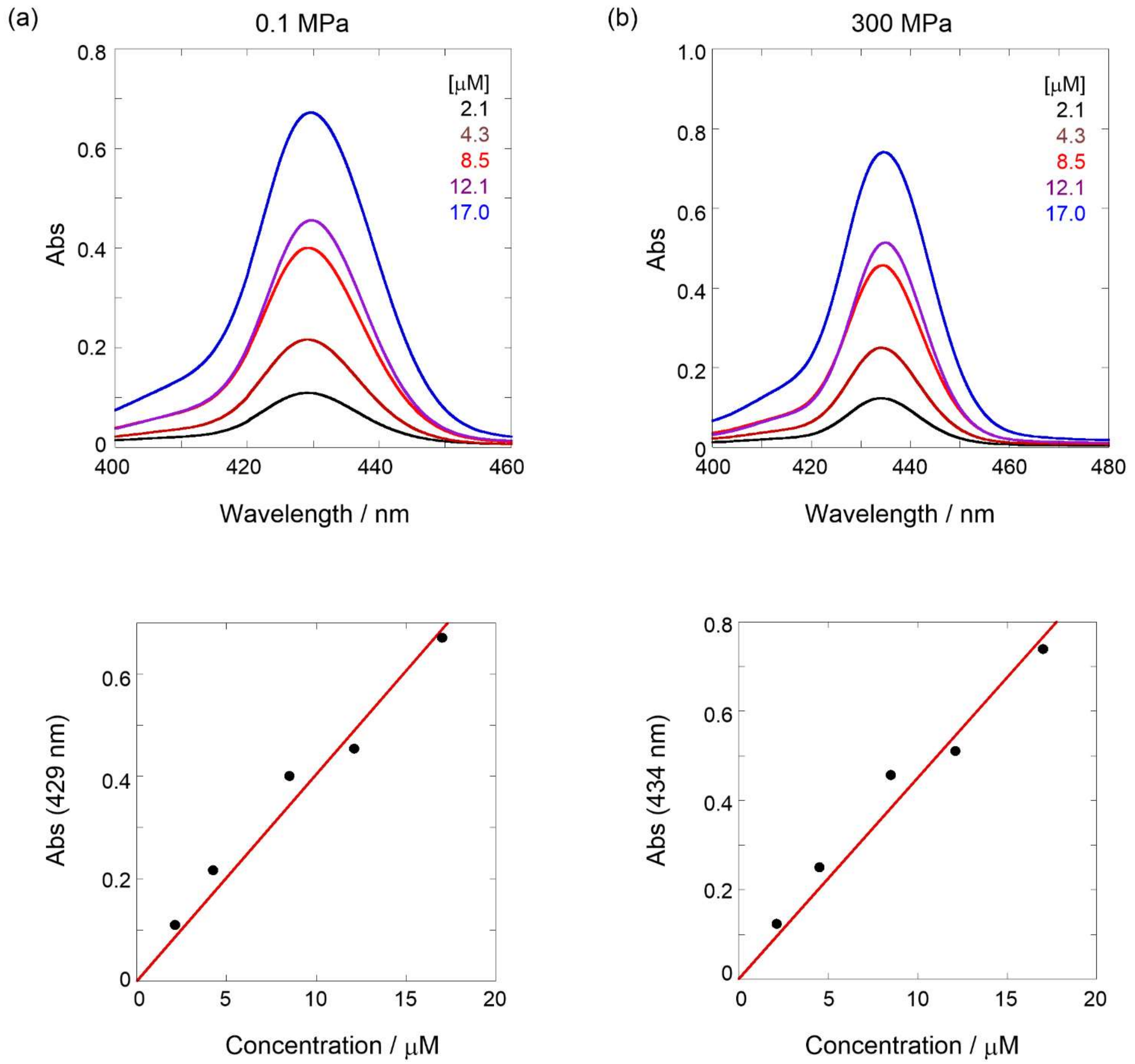

Figure S4. Concentration-dependent UV/vis spectra (top) and plots of the absorbance maxima (bottom) of **Por-Cy** (2.1–17.0 μM) in toluene at (a) 0.1 MPa (r = 0.991) and (b) 300 MPa (r = 0.989).

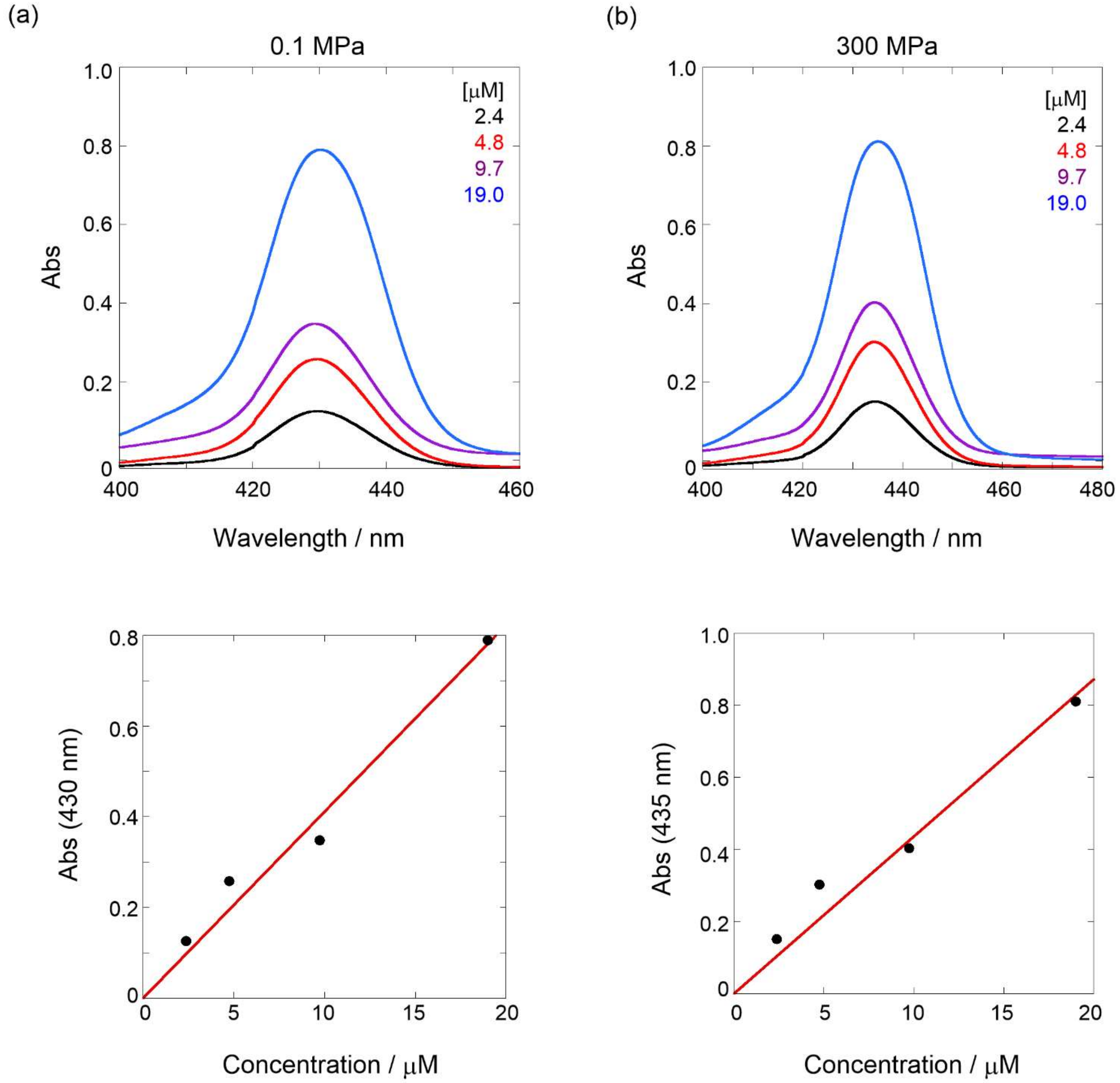

Figure S5. Concentration-dependent UV/vis spectra (top) and plots of the absorbance maxima (bottom) of **Por-DPhEt** (2.4–19.0 μM) in toluene at (a) 0.1 MPa (r = 0.988) and (b) 300 MPa (r = 0.991).

Wavelength Shifts under High Pressures

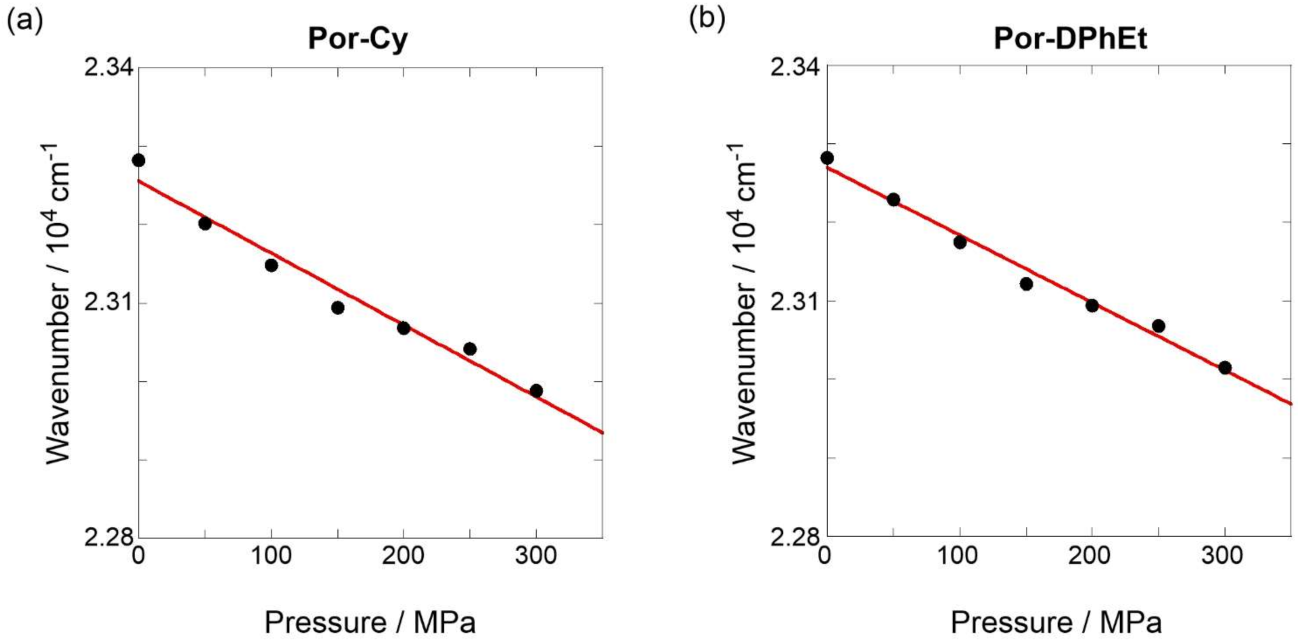

Figure S6. Absorption maxima around the Soret band as a function of pressure for (a) **Por-Cy** (r = 0.984) and (b) **Por-DPhEt** (r = 0.992).

Fluorescence Lifetime Decays

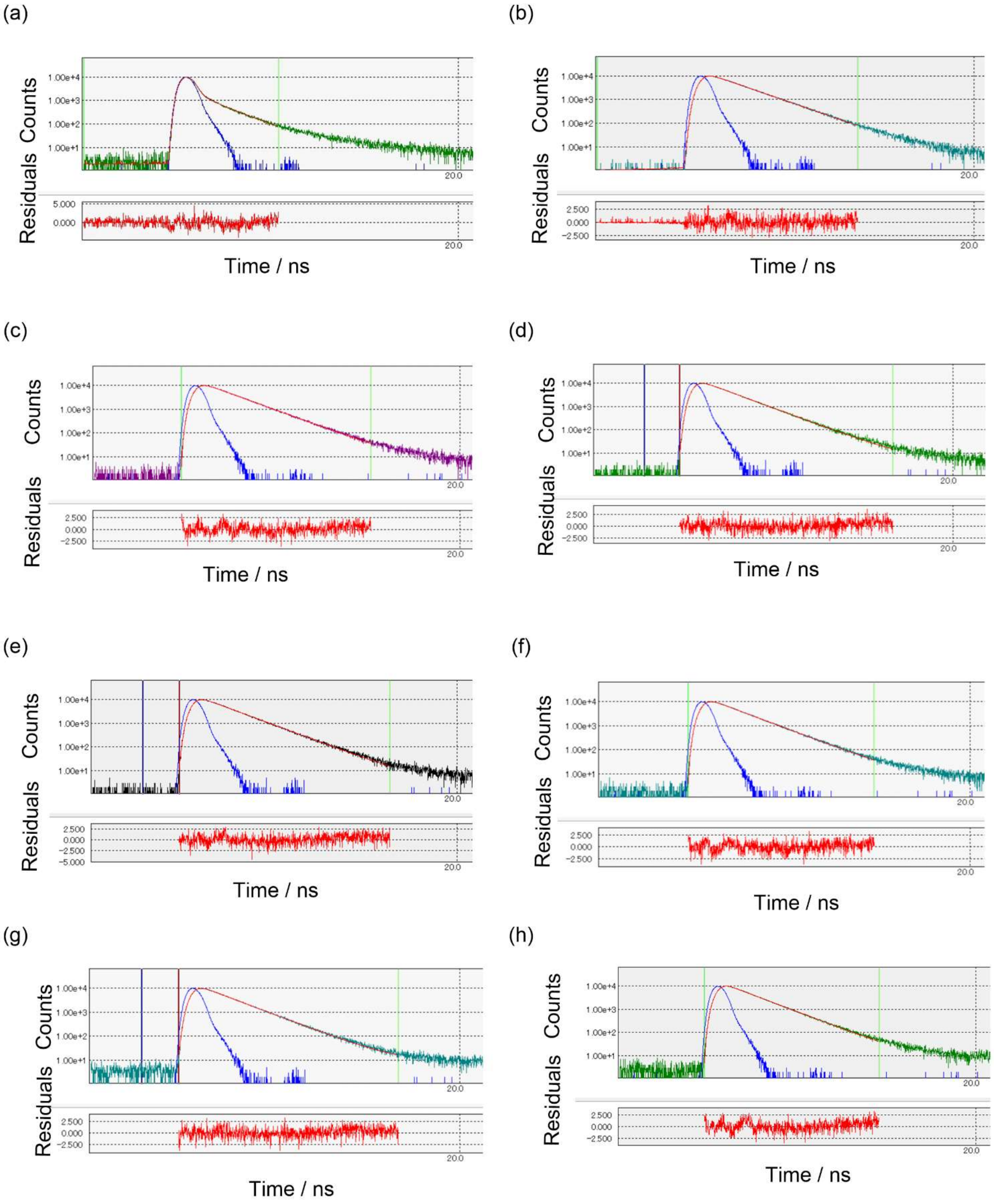

Figure S7. Time-correlated fluorescence decays of **Por-Cy** (12.1 μM) monitored at (a) 436 nm and (b) 605 nm at 0.1 MPa, (c) 605 nm at 50 MPa, (d) 605 nm at 100 MPa, (e) 605 nm at 150 MPa, (f) 605 nm at 200 MPa, (g) 605 nm at 250 MPa, and (h) 605 nm at 300 MPa in toluene at room temperature in a high-pressure cell. The colored, red, and blue lines represent the fluorescence decay, fitting result, and instrument response function, respectively.

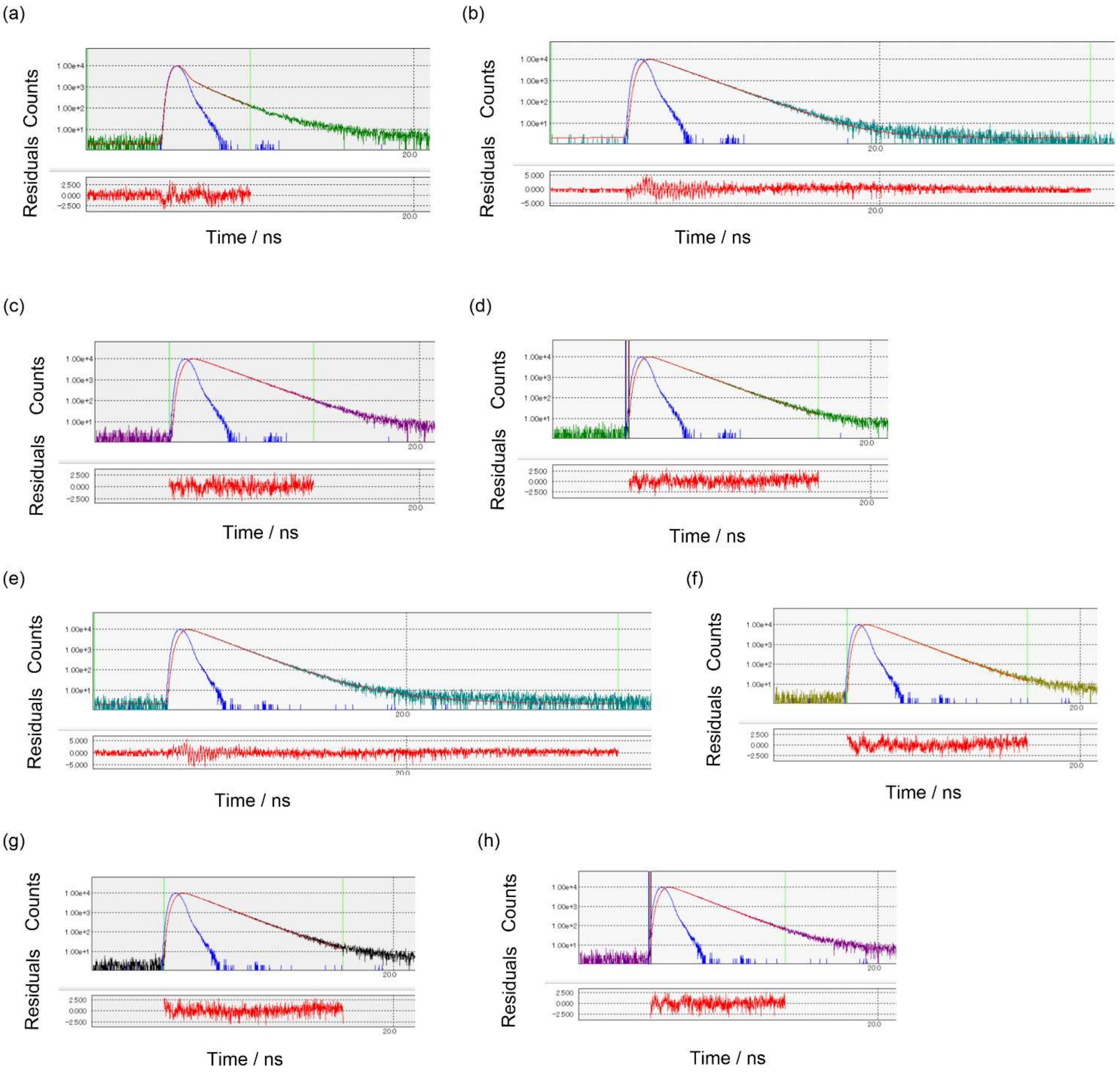

Figure S8. Time-correlated fluorescence decays of **Por-DPhEt** (9.7 μM) monitored at (a) 436 nm and (b) 605 nm at 0.1 MPa, (c) 605 nm at 50 MPa, (d) 605 nm at 100 MPa, (e) 605 nm at 150 MPa, (f) 605 nm at 200 MPa, (g) 605 nm at 250 MPa, and (h) 605 nm at 300 MPa in toluene at room temperature in a high-pressure cell. The colored, red, and blue lines represent the fluorescence decay, fitting result, and the instrument response function, respectively.

Summary of Structure Optimization

All computational studies were performed using Gaussian 16[2].

(1*S*,2*S*)-diphenylethane-1,2-dimethyl (**DPhEt**)

-56.1°

Symbol	X	Y	Z
C	2.02769	-1.882573	1.107891
C	2.833872	-2.06872	-0.010242
C	2.931303	-1.05581	-0.959999
C	2.228032	0.132062	-0.789688
C	1.415455	0.331836	0.329479
C	1.326597	-0.692351	1.272672
C	0.603165	1.604574	0.491313
C	-0.60245	1.604702	-0.491176
C	-1.415192	0.332238	-0.329406
C	-1.327349	-0.691633	-1.273008
C	-2.02903	-1.881544	-1.10833
C	-2.83476	-2.067664	0.010113
C	-2.931148	-1.055059	0.960328
C	-2.227311	0.132471	0.790117
H	1.939633	-2.667201	1.852924
H	3.381238	-2.996632	-0.142383
H	3.556832	-1.190851	-1.837192
H	2.310703	0.910795	-1.543199
H	0.682849	-0.561366	2.138073
H	0.187675	1.60264	1.508042
H	-0.186837	1.602608	-1.507858
H	-0.683938	-0.560693	-2.138668
H	-1.941761	-2.665945	-1.853695
H	-3.382585	-2.995318	0.142173

[2] Frisch, M. J.; Trucks, G. W.; Schlegel, H. B.; Scuseria, G. E.; Robb, M. A.; Cheeseman, J. R.; Scalmani, G.; Barone, V.; Petersson, G. A.; Nakatsuji, H.; Li, X.; Caricato, M.; Marenich, A. V.; Bloino, J.; Janesko, B. G.; Gomperts, R.; Mennucci, B.; Hratchian, H. P.; Ortiz, J. V.; Izmaylov, A. F.; Sonnenberg, J. L.; Williams-Young, D.; Ding, F.; Lipparini, F.; Egidi, F.; Goings, J.; Peng, B.; Petrone, A.; Henderson, T.; Ranasinghe, D.; Zakrzewski, V. G.; Gao, J. Rega, N.; Zheng, G.; Liang, W.; Hada, M.; Ehara, M.; Toyota, K.; Fukuda, R.; Hasegawa, J.; Ishida, M.; Nakajima, T.; Honda, Y.; Kitao, O.; Nakai, H.; Vreven, T.; Throssell, K.; Montgomery Jr., J. A.; Peralta, J. E.; Ogliaro, F.; Bearpark, M. J.; Heyd, J. J.; Brothers, E. N.; Kudin, K. N.; Staroverov, V. N.; Keith, T. A.; Kobayashi, R.; Normand, J.; Raghavachari, K.; Rendell, A. P.; Burant, J. C.; Iyengar, S. S.; Tomasi, J.; Cossi, M.; Millam, J. M.; Klene, M.; Adamo, C.; Cammi, R.; Ochterski, J. W.; Martin, R. L.; Morokuma, K.; Farkas, O.; Foresman, J. B.; Fox, D. J. Gaussian, Inc. **2016**, Wallingford CT.

H	-3.556307	-1.190112	1.837782
H	-2.309109	0.910958	1.54398
C	-1.475335	2.857134	-0.341819
H	-1.793719	3.001818	0.696202
H	-0.937813	3.757057	-0.651462
H	-2.373883	2.77271	-0.958606
C	1.476259	2.856826	0.341795
H	1.794478	3.001457	-0.696282
H	0.938862	3.756809	0.651479
H	2.374893	2.772335	0.958447

-36.1°

Symbol	X	Y	Z
C	2.167472	-1.977487	0.914983
C	3.145274	-1.944654	-0.073878
C	3.291826	-0.801417	-0.854759
C	2.467278	0.298699	-0.644194
C	1.485198	0.280925	0.35005
C	1.346337	-0.873312	1.12213
C	0.552462	1.459916	0.551616
C	-0.550796	1.460045	-0.551374
C	-1.484551	0.281797	-0.349752
C	-1.349058	-0.87131	-1.123929
C	-2.17158	-1.9746	-0.916932
C	-3.147308	-1.941843	0.073872
C	-3.290608	-0.799527	0.856844
C	-2.46484	0.299581	0.64638
H	2.039888	-2.865706	1.52597
H	3.787915	-2.804046	-0.236984
H	4.050657	-0.765881	-1.630519
H	2.589865	1.182149	-1.265171
H	0.572666	-0.9106	1.884135
H	0.034588	1.300676	1.507211
H	-0.032801	1.300088	-1.506788
H	-0.576972	-0.908647	-1.887535
H	-2.046445	-2.862028	-1.52957
H	-3.791033	-2.800426	0.23696
H	-4.047927	-0.76415	1.634091
H	-2.584795	1.18232	1.268911
C	-1.294472	2.794209	-0.680917
H	-1.74311	3.109904	0.267538
H	-0.622254	3.589922	-1.010348

H	-2.099724	2.704935	-1.41503
C	1.296848	2.793706	0.680219
H	1.744484	3.109186	-0.268793
H	0.625209	3.589606	1.010313
H	2.102898	2.704326	1.413445

-16.1°

Symbol	X	Y	Z
C	2.429377	-2.017351	0.716945
C	3.493156	-1.767558	-0.143455
C	3.599462	-0.522703	-0.758656
C	2.649178	0.462005	-0.51205
C	1.579315	0.226639	0.356668
C	1.482585	-1.026855	0.963218
C	0.512379	1.276695	0.595608
C	-0.512456	1.276662	-0.595623
C	-1.579311	0.226548	-0.356599
C	-1.482258	-1.027125	-0.962923
C	-2.428919	-2.01765	-0.716699
C	-3.493013	-1.76781	0.14341
C	-3.599648	-0.522897	0.758287
C	-2.649391	0.46194	0.511711
H	2.334123	-2.985388	1.199092
H	4.234499	-2.536834	-0.335189
H	4.425202	-0.3183	-1.433358
H	2.740412	1.42602	-1.005379
H	0.647833	-1.230105	1.628375
H	-0.051288	0.952773	1.479587
H	0.051117	0.952795	-1.479675
H	-0.647268	-1.230326	-1.627793
H	-2.333483	-2.985804	-1.198576
H	-4.234281	-2.537194	0.335004
H	-4.425559	-0.318361	1.432736
H	-2.740956	1.426007	1.004884
C	-1.083904	2.651013	-0.961532
H	-1.639819	3.111258	-0.137912
H	-0.288595	3.341844	-1.251138
H	-1.771169	2.551209	-1.806128
C	1.083589	2.651086	0.961658
H	1.639898	3.111256	0.138272
H	0.288066	3.341885	1.250774
H	1.770432	2.55143	1.806618

3.9°

Symbol	X	Y	Z
C	3.123536	-1.833908	0.744585
C	4.040103	-1.393186	-0.20334
C	3.803069	-0.200876	-0.884014
C	2.659618	0.541201	-0.613625
C	1.731085	0.111032	0.340132
C	1.979222	-1.085761	1.011662
C	0.479022	0.918994	0.625995
C	-0.478911	0.918857	-0.626041
C	-1.731008	0.110988	-0.340077
C	-1.97958	-1.085515	-1.011962
C	-3.124002	-1.833489	-0.744866
C	-4.04024	-1.392883	0.203432
C	-3.80277	-0.200863	0.88446
C	-2.659214	0.541043	0.614048
H	3.296397	-2.76339	1.278354
H	4.932682	-1.973894	-0.413341
H	4.511692	0.150948	-1.627593
H	2.48195	1.467411	-1.155096
H	1.264603	-1.438572	1.750565
H	-0.053343	0.400193	1.430047
H	0.053469	0.399923	-1.429997
H	-1.265217	-1.438237	-1.751155
H	-3.297202	-2.762752	-1.278907
H	-4.932897	-1.973465	0.413452
H	-4.511134	0.150868	1.62833
H	-2.481203	1.467036	1.155782
C	-0.83931	2.306151	-1.174856
H	-1.399941	2.905637	-0.451789
H	0.054946	2.869329	-1.457114
H	-1.463884	2.197088	-2.065726
C	0.839385	2.306385	1.174566
H	1.399984	2.905792	0.451412
H	-0.05489	2.869579	1.45673
H	1.463962	2.197488	2.065454

23.9°

Symbol	X	Y	Z
C	3.346287	-1.829175	0.47358
C	4.303791	-1.103819	-0.227485

C	4.014874	0.196225	-0.633326
C	2.780187	0.764278	-0.338035
C	1.807972	0.046932	0.366225
C	2.111282	-1.256515	0.764465
C	0.436451	0.633109	0.655619
C	-0.436469	0.633173	-0.655591
C	-1.807976	0.046949	-0.366229
C	-2.111214	-1.256522	-0.764428
C	-3.346206	-1.829236	-0.473559
C	-4.30377	-1.103893	0.227422
C	-4.014941	0.196202	0.633187
C	-2.780275	0.764301	0.337922
H	3.559905	-2.843356	0.797018
H	5.268279	-1.546399	-0.4552
H	4.754981	0.771788	-1.180885
H	2.574191	1.780889	-0.660896
H	1.366949	-1.830809	1.310181
H	-0.055055	-0.047399	1.35826
H	0.055047	-0.047261	-1.358297
H	-1.36684	-1.830815	-1.31009
H	-3.559749	-2.843444	-0.796962
H	-5.26825	-1.546499	0.455124
H	-4.755115	0.771759	1.180663
H	-2.574353	1.780952	0.660701
C	-0.549501	1.996775	-1.34601
H	-1.035823	2.744095	-0.713964
H	0.436791	2.383724	-1.620944
H	-1.138507	1.901466	-2.262137
C	0.549504	1.996635	1.346193
H	1.035912	2.743997	0.714264
H	-0.436784	2.383613	1.621104
H	1.138444	1.901178	2.262347

43.9°

Symbol	X	Y	Z
C	3.50675	-1.765952	0.027775
C	4.480921	-0.812878	-0.257259
C	4.14511	0.535957	-0.22499
C	2.846831	0.930429	0.089335
C	1.857847	-0.013595	0.37518
C	2.212557	-1.366048	0.33929
C	0.413058	0.364301	0.666282

C	-0.413051	0.363703	-0.666511
C	-1.857881	-0.013868	-0.375221
C	-2.212964	-1.366225	-0.339348
C	-3.507173	-1.765768	-0.027451
C	-4.480982	-0.812443	0.258001
C	-4.14481	0.536296	0.225709
C	-2.846507	0.930414	-0.088989
H	3.756615	-2.82239	0.011961
H	5.493551	-1.119793	-0.498717
H	4.896696	1.288711	-0.442516
H	2.610735	1.989529	0.11462
H	1.457064	-2.116173	0.562175
H	-0.00322	-0.439746	1.283695
H	0.00324	-0.440913	-1.283174
H	-1.457766	-2.116545	-0.562551
H	-3.757343	-2.822134	-0.011645
H	-5.49361	-1.119106	0.499784
H	-4.896102	1.289254	0.443538
H	-2.610129	1.989452	-0.114329
C	-0.286583	1.665431	-1.459206
H	-0.68379	2.527475	-0.917912
H	0.763776	1.872977	-1.686422
H	-0.828573	1.587559	-2.405483
C	0.286782	1.666777	1.457788
H	0.683203	2.528374	0.915208
H	-0.763423	1.874163	1.685797
H	0.829657	1.590115	2.40366

63.9°

Symbol	X	Y	Z
C	3.721486	-1.562669	-0.236067
C	4.60353	-0.485371	-0.253213
C	4.131065	0.787912	0.044587
C	2.787819	0.984735	0.356132
C	1.890869	-0.085192	0.37527
C	2.382371	-1.359626	0.075426
C	0.408586	0.083568	0.66612
C	-0.408704	0.084364	-0.666398
C	-1.890916	-0.0847	-0.375534
C	-2.382082	-1.359432	-0.076123
C	-3.721047	-1.562917	0.235468
C	-4.603403	-0.485809	0.253139

C	-4.131327	0.787642	-0.044305
C	-2.788114	0.984929	-0.355962
H	4.078345	-2.563126	-0.460595
H	5.650805	-0.638821	-0.493227
H	4.809825	1.635261	0.036986
H	2.440593	1.986549	0.587405
H	1.699982	-2.206697	0.091422
H	0.09189	-0.81746	1.207122
H	-0.091985	-0.816099	-1.208338
H	-1.699442	-2.206293	-0.092536
H	-4.077697	-2.563524	0.459665
H	-5.650578	-0.6397	0.493305
H	-4.81023	1.634872	-0.036335
H	-2.441284	1.98694	-0.586945
C	-0.085488	1.279893	-1.564124
H	-0.297423	2.237743	-1.079936
H	0.97405	1.276259	-1.831963
H	-0.67264	1.236892	-2.485536
C	0.085371	1.278092	1.565212
H	0.296476	2.236512	1.081758
H	-0.973975	1.273725	1.833797
H	0.673195	1.234463	2.486171

83.9°

Symbol	X	Y	Z
C	3.907627	-1.312882	-0.343481
C	4.643765	-0.136746	-0.226684
C	4.010782	1.025582	0.200439
C	2.65225	1.014439	0.505637
C	1.901261	-0.156806	0.390858
C	2.55213	-1.318415	-0.035449
C	0.410922	-0.20254	0.667722
C	-0.410848	-0.20234	-0.66769
C	-1.901166	-0.156632	-0.390701
C	-2.552005	-1.318364	0.035334
C	-3.907541	-1.312993	0.343216
C	-4.643754	-0.136901	0.226521
C	-4.010803	1.025541	-0.200349
C	-2.652229	1.014566	-0.505372
H	4.391392	-2.228506	-0.669501
H	5.70304	-0.128276	-0.462876
H	4.575599	1.947646	0.298187

H	2.174864	1.931535	0.836023
H	1.98408	-2.241705	-0.123906
H	0.212345	-1.178525	1.13103
H	-0.212253	-1.178236	-1.131207
H	-1.983907	-2.241635	0.123684
H	-4.391279	-2.228709	0.669025
H	-5.703058	-0.128549	0.462585
H	-4.575691	1.947568	-0.29803
H	-2.17489	1.931751	-0.835574
C	0.080196	0.8469	-1.668672
H	0.092468	1.855324	-1.239652
H	1.098541	0.619927	-1.988681
H	-0.564723	0.866345	-2.551721
C	-0.080436	0.846536	1.66874
H	-0.09327	1.854917	1.239625
H	-1.098667	0.619129	1.98882
H	0.564513	0.866327	2.551756

103.9°

Symbol	X	Y	Z
C	4.066682	-1.05936	-0.307085
C	4.611805	0.216816	-0.196369
C	3.803388	1.271962	0.21384
C	2.460121	1.054578	0.506747
C	1.898597	-0.219229	0.396365
C	2.724777	-1.270877	-0.010157
C	0.430378	-0.483864	0.664169
C	-0.430268	-0.484399	-0.664023
C	-1.898467	-0.219597	-0.395965
C	-2.724759	-1.270897	0.010986
C	-4.066759	-1.059129	0.30762
C	-4.611726	0.216978	0.196294
C	-3.803141	1.271894	-0.214376
C	-2.459922	1.05427	-0.507004
H	4.688776	-1.892444	-0.619606
H	5.659485	0.386083	-0.423653
H	4.219187	2.270511	0.307789
H	1.843651	1.889573	0.825176
H	2.30544	-2.270653	-0.095517
H	0.374309	-1.514352	1.038059
H	-0.374129	-1.515172	-1.03715
H	-2.305649	-2.27073	0.096798

H	-4.688875	-1.892081	0.620442
H	-5.659398	0.386571	0.423373
H	-4.218939	2.270403	-0.308763
H	-1.843259	1.888997	-0.825746
C	0.158405	0.379221	-1.785357
H	0.298802	1.421816	-1.479356
H	1.13621	0.001719	-2.088604
H	-0.50504	0.369346	-2.654881
C	-0.158872	0.380501	1.784599
H	-0.299401	1.422875	1.477949
H	-1.136783	0.003025	2.087585
H	0.504189	0.371315	2.654415

123.9°

Symbol	X	Y	Z
C	4.199091	-0.696462	-0.16053
C	4.43733	0.673633	-0.178524
C	3.389097	1.552961	0.07891
C	2.116039	1.06424	0.350549
C	1.859374	-0.310138	0.368178
C	2.921837	-1.17883	0.111236
C	0.47046	-0.860268	0.642576
C	-0.470466	-0.860815	-0.641832
C	-1.85937	-0.310467	-0.367865
C	-2.922004	-1.178907	-0.110827
C	-4.19926	-0.696242	0.16046
C	-4.437302	0.673888	0.177861
C	-3.388883	1.55297	-0.079698
C	-2.115845	1.063962	-0.350859
H	5.008721	-1.393126	-0.355179
H	5.431814	1.054179	-0.388503
H	3.563722	2.624503	0.069367
H	1.309489	1.764818	0.548019
H	2.744506	-2.251433	0.124038
H	0.610483	-1.916069	0.900049
H	-0.610421	-1.916815	-0.898507
H	-2.744821	-2.251539	-0.12316
H	-5.009034	-1.39271	0.355203
H	-5.431773	1.054665	0.387481
H	-3.563369	2.624539	-0.070613
H	-1.309127	1.764316	-0.548455
C	0.144431	-0.205156	-1.885923

H	0.358855	0.856402	-1.738194
H	1.087445	-0.686053	-2.156787
H	-0.547465	-0.2968	-2.727941
C	-0.144512	-0.20385	1.886227
H	-0.358686	0.857689	1.738027
H	-1.087674	-0.684441	2.157128
H	0.547237	-0.295237	2.728395

143.9°

Symbol	X	Y	Z
C	4.109719	-0.307892	-0.248332
C	4.114986	1.078233	-0.135187
C	2.946503	1.736765	0.237188
C	1.785201	1.01502	0.492554
C	1.761293	-0.378454	0.374598
C	2.943114	-1.023604	0.004692
C	0.495178	-1.186167	0.620028
C	-0.49519	-1.186378	-0.619596
C	-1.761291	-0.378575	-0.374417
C	-2.943223	-1.023593	-0.004663
C	-4.109824	-0.307751	0.248052
C	-4.114952	1.078357	0.134754
C	-2.946335	1.736758	-0.237459
C	-1.785052	1.014892	-0.492528
H	5.015173	-0.835305	-0.532367
H	5.022057	1.640957	-0.331373
H	2.939173	2.818262	0.333138
H	0.885643	1.547035	0.786771
H	2.948099	-2.106893	-0.088415
H	0.818228	-2.225846	0.743422
H	-0.818249	-2.226094	-0.742647
H	-2.948323	-2.106869	0.088569
H	-5.015373	-0.835062	0.531977
H	-5.022011	1.641183	0.330706
H	-2.938899	2.818246	-0.33351
H	-0.885373	1.546784	-0.786603
C	0.163618	-0.751298	-1.934694
H	0.524617	0.278259	-1.894134
H	1.026607	-1.381765	-2.168828
H	-0.554751	-0.832588	-2.755188
C	-0.163708	-0.750694	1.934956
H	-0.524832	0.278803	1.894045

H	-1.026644	-1.381175	2.169254
H	0.554632	-0.831642	2.755508

163.9°

Symbol	X	Y	Z
C	3.928318	0.028239	-0.37327
C	3.782831	1.378446	-0.071329
C	2.580915	1.831754	0.463643
C	1.53347	0.944328	0.691795
C	1.659508	-0.413642	0.381846
C	2.876276	-0.853235	-0.146445
C	0.521901	-1.40235	0.588852
C	-0.522027	-1.402697	-0.587998
C	-1.659545	-0.413796	-0.381567
C	-2.876701	-0.853126	0.146106
C	-3.928642	0.028583	0.372343
C	-3.782676	1.378781	0.070467
C	-2.580397	1.83181	-0.463861
C	-1.533024	0.944117	-0.691458
H	4.863043	-0.341518	-0.783446
H	4.600665	2.070435	-0.245411
H	2.457616	2.881811	0.710843
H	0.606364	1.318975	1.112771
H	2.999471	-1.906611	-0.38576
H	0.976758	-2.400383	0.574856
H	-0.976906	-2.400707	-0.57343
H	-3.000211	-1.906477	0.38536
H	-4.863698	-0.340909	0.782003
H	-4.60047	2.070919	0.244143
H	-2.456678	2.881833	-0.710994
H	-0.605613	1.318568	-1.111943
C	0.137886	-1.204798	-1.956272
H	0.625878	-0.231412	-2.03826
H	0.907889	-1.963896	-2.129266
H	-0.607277	-1.285677	-2.752355
C	-0.138055	-1.203705	1.956979
H	-0.626144	-0.23032	2.038402
H	-0.907998	-1.962778	2.130369
H	0.60708	-1.284098	2.753136

183.9°

Symbol	X	Y	Z

C	3.684248	0.355186	-0.511736
C	3.439585	1.627255	-0.004103
C	2.262271	1.867598	0.697609
C	1.336302	0.84654	0.888232
C	1.563831	-0.435387	0.377927
C	2.754694	-0.661584	-0.318282
C	0.559654	-1.562638	0.546563
C	-0.55958	-1.562554	-0.546752
C	-1.563808	-0.435363	-0.377956
C	-2.754596	-0.661668	0.318349
C	-3.68421	0.355029	0.511901
C	-3.439693	1.627126	0.004268
C	-2.262462	1.867577	-0.697541
C	-1.336426	0.846595	-0.888251
H	4.601584	0.151471	-1.055482
H	4.162653	2.423554	-0.149797
H	2.061875	2.85513	1.101754
H	0.421695	1.057385	1.432091
H	2.954537	-1.653379	-0.716088
H	1.111263	-2.498612	0.388026
H	-1.111169	-2.498573	-0.388389
H	-2.954337	-1.65349	0.716135
H	-4.601487	0.151237	1.055716
H	-4.162822	2.423357	0.150031
H	-2.062181	2.855131	-1.101688
H	-0.42187	1.057518	-1.432165
C	0.027407	-1.610777	-1.96028
H	0.639102	-0.732399	-2.180592
H	0.669337	-2.490241	-2.077613
H	-0.769956	-1.667601	-2.70616
C	-0.02726	-1.611111	1.960109
H	-0.638845	-0.73272	2.180666
H	-0.669271	-2.490538	2.077293
H	0.770147	-1.668175	2.705923

203.9°

Symbol	X	Y	Z
C	3.359865	0.684019	-0.66079
C	3.053774	1.848467	0.035668
C	1.96184	1.862597	0.898989
C	1.180135	0.724187	1.060609
C	1.472758	-0.453086	0.365228

C	2.576393	-0.454041	-0.491631
C	0.609839	-1.6934	0.490582
C	-0.610022	-1.693263	-0.490456
C	-1.4727	-0.452782	-0.365111
C	-2.576983	-0.453862	0.490925
C	-3.360381	0.684262	0.659929
C	-3.053569	1.848914	-0.03588
C	-1.960948	1.863195	-0.898325
C	-1.179313	0.724712	-1.059791
H	4.212499	0.657505	-1.332345
H	3.663227	2.737809	-0.090337
H	1.712923	2.766398	1.446414
H	0.320206	0.760345	1.721605
H	2.826241	-1.363253	-1.032873
H	1.232182	-2.540782	0.173588
H	-1.232533	-2.540497	-0.1734
H	-2.827372	-1.36323	1.031656
H	-4.213519	0.65765	1.330841
H	-3.662965	2.738312	0.089994
H	-1.711432	2.76716	-1.445207
H	-0.318863	0.760995	-1.720108
C	-0.17036	-1.988606	-1.927385
H	0.548913	-1.249523	-2.293886
H	0.310332	-2.970249	-1.981978
H	-1.02834	-1.987608	-2.605181
C	0.169867	-1.988607	1.927446
H	-0.549912	-1.249779	2.29349
H	-0.310375	-2.970457	1.982109
H	1.027594	-1.987037	2.605565

223.9°

Symbol	X	Y	Z
C	2.979232	0.987197	-0.880321
C	2.711276	2.022035	0.009145
C	1.803589	1.817112	1.044875
C	1.165566	0.590546	1.185729
C	1.420092	-0.456842	0.296114
C	2.340039	-0.240614	-0.731997
C	0.687887	-1.780006	0.382442
C	-0.686888	-1.78005	-0.3827
C	-1.41972	-0.45724	-0.29607
C	-2.339845	-0.24172	0.732024

C	-2.979863	0.985654	0.880447
C	-2.712568	2.020754	-0.008908
C	-1.804685	1.816544	-1.044609
C	-1.16585	0.590415	-1.185564
H	3.690198	1.131928	-1.688044
H	3.207599	2.981013	-0.101383
H	1.585195	2.619254	1.742951
H	0.439919	0.457043	1.981701
H	2.558448	-1.047785	-1.427135
H	1.316091	-2.510178	-0.144073
H	-1.314869	-2.510665	0.143482
H	-2.557766	-1.049109	1.427062
H	-3.690962	1.129824	1.688152
H	-3.209552	2.979382	0.101682
H	-1.586785	2.618905	-1.742586
H	-0.440037	0.457501	-1.981478
C	-0.521513	-2.31405	-1.80819
H	0.196876	-1.718482	-2.382526
H	-0.150895	-3.343035	-1.784131
H	-1.473137	-2.301643	-2.346886
C	0.522909	-2.31455	1.807763
H	-0.195552	-1.71941	2.382445
H	0.152637	-3.34365	1.78342
H	1.474641	-2.302011	2.346269

243.9°

Symbol	X	Y	Z
C	2.740757	1.220665	-1.068446
C	2.573967	2.121171	-0.02267
C	1.839579	1.7409	1.097943
C	1.275315	0.473493	1.168528
C	1.426077	-0.43928	0.12061
C	2.171461	-0.047725	-0.991982
C	0.774949	-1.807966	0.150164
C	-0.77569	-1.807781	-0.150114
C	-1.426318	-0.43886	-0.12064
C	-2.171392	-0.046896	0.992012
C	-2.74011	1.221757	1.068452
C	-2.573039	2.122111	0.022592
C	-1.838946	1.741435	-1.098078
C	-1.275252	0.473774	-1.168636
H	3.315031	1.502899	-1.945595

H	3.01309	3.112247	-0.078102
H	1.700738	2.43759	1.918788
H	0.686368	0.20214	2.039096
H	2.302664	-0.7459	-1.815378
H	1.243106	-2.382306	-0.658647
H	-1.243983	-2.381872	0.658791
H	-2.302806	-0.744954	1.815474
H	-3.314156	1.504307	1.945648
H	-3.011717	3.113385	0.077997
H	-1.6999	2.438005	-1.91899
H	-0.686533	0.202094	-2.039257
C	-1.065628	-2.56863	-1.448817
H	-0.580492	-2.092178	-2.30752
H	-0.685237	-3.59301	-1.382126
H	-2.139626	-2.611841	-1.649946
C	1.064545	-2.56873	1.449002
H	0.57934	-2.092054	2.307548
H	0.683954	-3.593039	1.382372
H	2.138498	-2.612135	1.650332

263.9°

Symbol	X	Y	Z
C	2.794514	1.360003	-1.040293
C	2.714327	2.112277	0.125492
C	2.001817	1.614709	1.214464
C	1.377811	0.376709	1.13452
C	1.443802	-0.387515	-0.034339
C	2.16278	0.120804	-1.115158
C	0.773657	-1.746365	-0.125511
C	-0.774321	-1.746283	0.125578
C	-1.444077	-0.387251	0.034286
C	-2.162495	0.121608	1.115232
C	-2.793667	1.361085	1.04033
C	-2.713473	2.113105	-0.125623
C	-2.001513	1.615011	-1.214708
C	-1.378045	0.376734	-1.134718
H	3.34698	1.736494	-1.895768
H	3.200866	3.080534	0.187123
H	1.928848	2.196615	2.127996
H	0.811271	0.008449	1.985343
H	2.225873	-0.459817	-2.032432
H	0.94204	-2.119008	-1.143272

H	-0.942719	-2.118722	1.143405
H	-2.225582	-0.458825	2.032625
H	-3.345697	1.738013	1.895896
H	-3.199576	3.08158	-0.187273
H	-1.928546	2.196705	-2.128374
H	-0.811901	0.008085	-1.985637
C	-1.453911	-2.714082	-0.854936
H	-1.352282	-2.353659	-1.88329
H	-1.001484	-3.710425	-0.800995
H	-2.520922	-2.810218	-0.636299
C	1.452993	-2.714125	0.85525
H	1.351223	-2.353463	1.883516
H	1.000408	-3.710404	0.801408
H	2.520028	-2.810482	0.636827

283.9°

Symbol	X	Y	Z
C	2.794514	1.360003	-1.040293
C	2.714327	2.112277	0.125492
C	2.001817	1.614709	1.214464
C	1.377811	0.376709	1.13452
C	1.443802	-0.387515	-0.034339
C	2.16278	0.120804	-1.115158
C	0.773657	-1.746365	-0.125511
C	-0.774321	-1.746283	0.125578
C	-1.444077	-0.387251	0.034286
C	-2.162495	0.121608	1.115232
C	-2.793667	1.361085	1.04033
C	-2.713473	2.113105	-0.125623
C	-2.001513	1.615011	-1.214708
C	-1.378045	0.376734	-1.134718
H	3.34698	1.736494	-1.895768
H	3.200866	3.080534	0.187123
H	1.928848	2.196615	2.127996
H	0.811271	0.008449	1.985343
H	2.225873	-0.459817	-2.032432
H	0.94204	-2.119008	-1.143272
H	-0.942719	-2.118722	1.143405
H	-2.225582	-0.458825	2.032625
H	-3.345697	1.738013	1.895896
H	-3.199576	3.08158	-0.187273
H	-1.928546	2.196705	-2.128374

H	-0.811901	0.008085	-1.985637
C	-1.453911	-2.714082	-0.854936
H	-1.352282	-2.353659	-1.88329
H	-1.001484	-3.710425	-0.800995
H	-2.520922	-2.810218	-0.636299
C	1.452993	-2.714125	0.85525
H	1.351223	-2.353463	1.883516
H	1.000408	-3.710404	0.801408
H	2.520028	-2.810482	0.636827

303.9°

Symbol	X	Y	Z
C	2.028654	1.881979	-1.108157
C	2.834349	2.068214	0.010289
C	2.931036	1.055537	0.960392
C	2.2275	-0.132161	0.790104
C	1.415359	-0.332011	-0.329376
C	1.327271	0.691905	-1.272908
C	0.602843	-1.604632	-0.491247
C	-0.602807	-1.604642	0.491202
C	-1.415359	-0.332044	0.329346
C	-1.327295	0.691849	1.272907
C	-2.0287	1.881912	1.108186
C	-2.834403	2.068156	-0.010255
C	-2.931059	1.055505	-0.960387
C	-2.227493	-0.132179	-0.790135
H	1.941167	2.666405	-1.853468
H	3.381936	2.995996	0.142437
H	3.556209	1.19066	1.837826
H	2.309588	-0.910703	1.543877
H	0.683887	0.560873	-2.138573
H	0.187347	-1.602588	-1.507976
H	-0.187271	-1.602566	1.507912
H	-0.683906	0.560809	2.138569
H	-1.941233	2.666318	1.85352
H	-3.382016	2.995925	-0.142381
H	-3.556227	1.190641	-1.837823
H	-2.309544	-0.910695	-1.54394
C	-1.475801	-2.856964	0.341813
H	-1.794426	-3.001507	-0.696145
H	-0.938262	-3.756968	0.651213
H	-2.374209	-2.772598	0.95882

C	1.475873	-2.856931	-0.341792
H	1.794186	-3.001622	0.696244
H	0.938485	-3.756919	-0.651494
H	2.374466	-2.772405	-0.958507
